# An Eight-Parent Multiparent Advanced Generation Inter-Cross Population for Winter-Sown Wheat: Creation, Properties, and Validation

**DOI:** 10.1534/g3.114.012963

**Published:** 2014-09-01

**Authors:** Ian J. Mackay, Pauline Bansept-Basler, Toby Barber, Alison R. Bentley, James Cockram, Nick Gosman, Andy J. Greenland, Richard Horsnell, Rhian Howells, Donal M. O’Sullivan, Gemma A. Rose, Phil J. Howell

**Affiliations:** * The John Bingham Laboratory, National Institute of Agricultural Botany (NIAB), Cambridge, CB3 0LE, United Kingdom

**Keywords:** quantitative genetics, genome-wide association scan, single nucleotide polymorphism, QTL mapping, recombinant inbred line (RIL), high-density genotyping, Multiparent Advanced Generation Inter-Cross (MAGIC), multiparental populations, MPP

## Abstract

MAGIC populations represent one of a new generation of crop genetic mapping resources combining high genetic recombination and diversity. We describe the creation and validation of an eight-parent MAGIC population consisting of 1091 F_7_ lines of winter-sown wheat (*Triticum aestivum* L.). Analyses based on genotypes from a 90,000-single nucleotide polymorphism (SNP) array find the population to be well-suited as a platform for fine-mapping quantitative trait loci (QTL) and gene isolation. Patterns of linkage disequilibrium (LD) show the population to be highly recombined; genetic marker diversity among the founders was 74% of that captured in a larger set of 64 wheat varieties, and 54% of SNPs segregating among the 64 lines also segregated among the eight founder lines. In contrast, a commonly used reference bi-parental population had only 54% of the diversity of the 64 varieties with 27% of SNPs segregating. We demonstrate the potential of this MAGIC resource by identifying a highly diagnostic marker for the morphological character "awn presence/absence" and independently validate it in an association-mapping panel. These analyses show this large, diverse, and highly recombined MAGIC population to be a powerful resource for the genetic dissection of target traits in wheat, and it is well-placed to efficiently exploit ongoing advances in phenomics and genomics. Genetic marker and trait data, together with instructions for access to seed, are available at http://www.niab.com/MAGIC/.

Recent advances in genotyping capabilities, especially in crops, mean that genetic marker density is no longer the limiting factor for QTL studies in plants. Accordingly, experimental design has re-focused on the levels of genetic recombination and diversity captured in mapping populations. The multiparent advanced generation inter-cross (MAGIC) population is one of a new generation of emerging mapping resources within plant genetics. In crops, the approach was first advocated in 2007 (Mackay and Powell 2007; Cavanagh *et al.* 2008). MAGIC populations are multi-founder equivalents of the advanced intercross introduced by Darvasi and Soller (1995) and are very similar to the heterogeneous stock and collaborative cross populations used in mouse genetics (Mott *et al.* 2000; Threadgill and Churchill 2012). They are created by several generations of intercrossing among multiple founder lines. Multiple founders contribute more allelic diversity than that captured in typical bi-parental mapping populations, whereas the multiple cycles of intercrossing give greater opportunities for recombination and, hence, greater precision in QTL location. MAGIC and MAGIC-like populations are now available in a range of plant and crop species, including Arabidopsis (Kover *et al.* 2009), rice (Bandillo *et al.* 2013) and barley (University of Bonn, J. Léon, personal communication). A four-founder Australian spring wheat MAGIC population has recently been described (Huang *et al.* 2012).

Here, we describe the creation and characterization of a large, genetically diverse eight-parent MAGIC population of direct relevance to UK and European winter wheat breeding. We exemplify the use of the population by investigating the genetic control of awning. Awns are bristle-like structures found on the ears of many cereal varieties, and awned wheat varieties are typically favored in drier climates. Three dominant awning inhibitors have been described in wheat, although none of the underlying genes has been cloned: *Hd* (*Hooded*, chromosome 4AS), *B1* (*Tipped 1*, 5AL), and *B2* (*Tipped 2*, 6BS) (McIntosh *et al.* 1998). Of the eight MAGIC population parents, one possesses awns (Soissons) but all others are awnless (Alchemy, Brompton, Claire, Hereward, Rialto, Robigus, Xi19). We map the wheat *B1* awning locus to a 7.5-cM interval, identifying a highly diagnostic marker for awn presence/absence, and validate in an independent association mapping population. We also describe the phenotypic data currently available for the population and those analyzed by Scutari *et al.* (2014).

## Materials and Methods

### MAGIC population construction

The eight founder varieties were selected in partnership with UK wheat breeders (Table 1). MAGIC population design was based around a simple replicated funnel crossing scheme of the form, {[(A×B) × (C×D)] × [(E×F) × (G×H)]} where the matched brackets (), [ ], and { } delineate the four two-way, two four-way, and one eight-way crosses, respectively, and the letters denote the eight founders, simplified here to "ABCDEFGH." Replicated schemes best sample diversity among the founders and reduce linkage disequilibrium (LD) within a MAGIC population; ideally, for an eight founder population (excluding reciprocal crosses) there are 28 possible F_1_ combinations, 210 four-way combinations (n(n-1)(n-2)(n-3)/8) among unrelated F_1_s, and 315 eight-way crosses (because the four-way cross ABCD can be paired with EFGH, EGFH, and EHFG). This design was used here, with the exception that two-thirds of the 315 possible eight-way crosses were made using each four-way twice. Individuals from these eight-way families were selfed through single seed descent (SSD) with a target population of 1000 recombinant inbred lines (RILs). The number of RILs contributed per family was kept as equal as possible within the constraints of variable seed set.

**Table 1:** Founder lines of the eight-parent MAGIC wheat population

Variety	Listing Year	Seed Yield (t/ha)*^a^*	NABIM Quality Group*^b^*	Trait Attributes
Alchemy	2006	9.163	4	Yield, disease resistance, breeding use, soft
Brompton	2005	9.151	4	Hard feed, 1BL/1RS, OWBM-resistant*^c^*
Claire	1999	8.654	3	Soft biscuit/distilling, slow apical development
Hereward	1991	7.683	1	High-quality benchmark 1 bread-making
Rialto	1994	8.377	2	Moderate bread-making, 1BL/1RS
Robigus	2003	9.053	3	Exotic introgression, disease resistance, breeding use, OWBM-resistant, *Rht-B1*
Soissons	1995	7.553	2	Bread-making quality, early flowering, *Rht-B1*
Xi19	2002	8.957	1	Bread-making quality, facultative type, breeding use

a Yield adjusted for site and year effects as estimated by Mackay *et al.* (2011).

b NABIM quality groups (http://www.nabim.com/): 1 (high bread-making); 2 (good bread-making); 3 (biscuit/cake); 4 (other).

c OWBM, orange wheat blossom midge.

### DNA extraction and genetic markers

Seedling leaf tissue for 720 F_5_ progeny bulks (10 F_5_ plants per line) of selfed F_4_ individuals were sampled, along with leaf tissue from all eight founder lines. Genomic DNA was extracted using a modified Tanksley extraction method (Fulton *et al.* 1995) and sent for SNP analysis (10 µg DNA per sample, adjusted to 100 ng/µl). Genotyping was performed using the Illumina Infinium iSelect 90,000 SNP wheat array (http://www.illumina.com/), provided as a service by the Department of Primary Industries (Victorian AgriBiosciences Center, Bundoora, VIC 3083, Australia). The development of this array and the corresponding consensus genetic map have been previously published (Wang *et al.* 2014). For unmapped SNPs, putative chromosomal allocation was determined by BLASTn searches of flow-sorted wheat chromosome arm genome survey sequence (http://www.wheatgenome.org/). Genetic diversity among the founders of the MAGIC population, a collection of 64 wheat varieties of predominantly UK origin, and an exemplar bi-parental population (the Avalon × Cadenza (A×C) doubled haploid mapping population; data from Snape *et al.* (2007)) were compared using publically available data (http://www.cerealsdb.uk.net) on the same Illumina Infinium iSelect 90,000 SNP array.

### MAGIC statistical analysis

Using mapped markers, LD heatmaps and LD decay plots were estimated using the R package, popgen (Marchini 2013). For display of genome-wide heatmaps, all chromosomes were concatenated, with their lengths first padded to 250 cM. Coefficients of LD were extracted and plotted against the genetic distance between pairs of markers. Smoothing curves were fitted to plots of LD decay by lowess, with a smoothing parameter of 0.1.

### Analysis of awning

The awn phenotype in the MAGIC population was scored as absence (score = 0) or presence (score = 1) among F_5_ families (selfed F_4_ progenies) grown in field multiplication plots in 2012. Association of line means with all markers was performed with the package lme4 (Bates *et al.* 2013) in R. Although the construction of the MAGIC population was designed to produce a population with uniform kinship relationships, some structure remains. Because the full pedigree of the population is known, this was accounted for using a mixed model with variance components to account for each stratum (between funnels and between outcrossed plants within funnels). Methods of analysis based on estimation of probabilities that an allele descends from each founder (Mott *et al.* 2000; Huang and George 2011) were not necessary to locate the major locus segregating for awning and have not been attempted.

Subsequently, the diagnostic SNP for awning was converted to the Kompetative Allele-Specific PCR (KASP) platform. KASP marker design and genotyping were performed by LGC Genomics (Hoddesdon, UK) and genotype data visualized using SNPviewer 1.3 (http:www.lgcgenomics.com/). The KASP marker was validated on an independent wheat association mapping (AM) panel consisting of 376 varieties genotyped with 1621 Diversity Array Technology (DArT) markers (data available at http://www.cerealsdb.uk.net/) and phenotyped for awn presence/absence (Supporting Information, Table S1). Data were analyzed using the mixed linear model (MLM) in efficient mixed-model association (EMMA) as implemented in the genome association and prediction integrated tool (GAPIT) in R (Lipka *et al.* 2012) using a marker-based kinship matrix derived within GAPIT from a subset of 927 markers giving a minimum inter-marker distance of 1 cM. This minimum distance prevents bias of the kinship matrix toward clusters of closely linked markers in small genomic regions (such as the 1BL/1RS translocation, polymorphic in European wheat). A Bonferroni corrected 1% significance threshold was used to account for multiple testing.

### Bioinformatic analysis

Flanking wheat genomic DNA sequences for SNPs present on the 90k array (Wang *et al.* 2014) were used as queries for BLAST searches against wheat genome survey sequence (GSS) generated from flow-sorted chromosome arms (http://wheat-urgi.versailles.inra.fr/) and predicted coding regions (CDS) from the genome assemblies of three sequenced Poaceae species using Phytozome v9.1 (http://www.phytozome.net/): *Oryza sativa* L. ssp. *japonica* cv. Nipponbare (MSU Rice Genome Annotation, release 7), *Brachypodium distachyon* L. accession Bd21 (JGI v1.0 8x assembly, MIPS/JGI v1.2 annotation), and *Sorghum bicolor* L. Moench accession BTx623 (release v1.0, Sbi1 assembly, Sbi1.4 gene set).

### Phenotype data

F_4_ multiplication nursery plots of all lines, each tracing a single F_3_ individual, were grown during 2010–2011 at the NIAB experimental farm in Cambridge, UK. These were assessed for flowering time using the breeders’ 1–9 scale (1 = very early; 9 = very late). Flowering time was calculated as the aggregate of five such scores taken at 3-d to 7-d intervals. Grain samples were hand-harvested from each plot to provide F_5_ pure seed (tracing single F_4_ plants), and the remainder of each plot was then harvested by plot combine to provide bulk F_5_ trial seed (tracing single F_3_ plants).

A large yield trial of F_5_ lines was grown during 2011–2012 at the same location. The trial was of 884 lines in two replicates, 85 lines with insufficient seed in one replicate, and 88 lines identified in 2011 as promising from a breeding perspective in three replicates. The eight founders plus an admixture bulk of all eight founders were also grown in three replicates. A commercial standard, the variety KWS Santiago, was grown in 12 replicates. The trial was designed using the freely available DEW software http://www.niab.com/dew/, with row and column blocks nested within main blocks. The total trial of 2156 plots (each 6 × 2 m) covered 2.6 ha.

F_5_ multiplication plots were also grown in an adjacent field during 2011-2012. Data on height (cm) and disease resistance to *Fusarium*, yellow rust, and mildew were scored on each plot. Disease levels were scored using the breeders’ 1–9 scale, where 1 = no infection and 9 = full infection (HGCA, 2012).

The eight founders and 1151 F_5_ lines were screened in an inoculated controlled environment experiment for seedling resistance to YR (*P. striiformis* f. sp. *tritici* "Warrior" race 11/08, virulent for *Yr1,2,3,4,6,7,9,17,32*, and on the varieties Spalding Prolific, Robigus, Solstice, Timber, Warrior). The susceptible cultivar Warrior was included as a positive control. Ten seedlings per line were grown in 96-well trays in two replicates under controlled environment conditions: long-day photoperiod (16 hr light/8 hr dark) and day/night temperature of 18°/11°. Five plates were grouped into larger blocks of size five. Disease assessment was conducted 18 d after inoculation using the 0–9 infection-type scoring system described by McNeal *et al.* (1971), where 0 represents immunity and 9 represents complete infection. An analysis of these data has been reported by Scutari *et al.* (2014).

## Results

### MAGIC population creation

Eight founder lines were selected on the basis of their seed yield, bread-making quality, disease resistance, and utility as parents within ongoing breeding programs (Table 1). Based on the optimized crossing design, all 28 F_1_ and 210 four-way crosses were made. However, only 210 of 315 eight-way crosses were made, due to practical constraints. From four-way onwards, a small number of crosses failed, and for these seed from the most closely related cross was substituted. Several extreme dwarf lines were eliminated during selfing and seed multiplication, because these cannot be satisfactorily grown and phenotyped under field conditions. These lines carry dwarfing alleles at both the major dwarfing loci *Reduced height-B1* (*Rht-B1*; from founder lines Robigus and Soissons) and *Rht-D1* (present in the remaining six founders). Currently, the MAGIC population numbers 1091 RILs at F_7_. Pedigree information for all MAGIC lines can be downloaded from http://www.niab.com/MAGIC/.

### MAGIC genotyping, allele frequencies, and LD

Illumina iSelect SNP scores on a panel of 64 wheat varieties are available from CerealsDB (http://www.cerealsdb.uk.net/). These 64 include the eight MAGIC founder lines and the two parents of the Avalon × Cadenza bi-parental mapping population that has been widely used in the UK. From this dataset 12,333 markers were classified as codominant, with a minor allele frequency (maf) ≥0.01 and with <10% missing data. Diversity (average expected heterozygosity under random mating) among these lines was 0.251. Among the eight founder lines, diversity was 0.186 with 6640 SNPs segregating. Between the two founders of the mapping population, diversity was 0.134 and 3313 SNPs were segregating.

**Figure 1:**
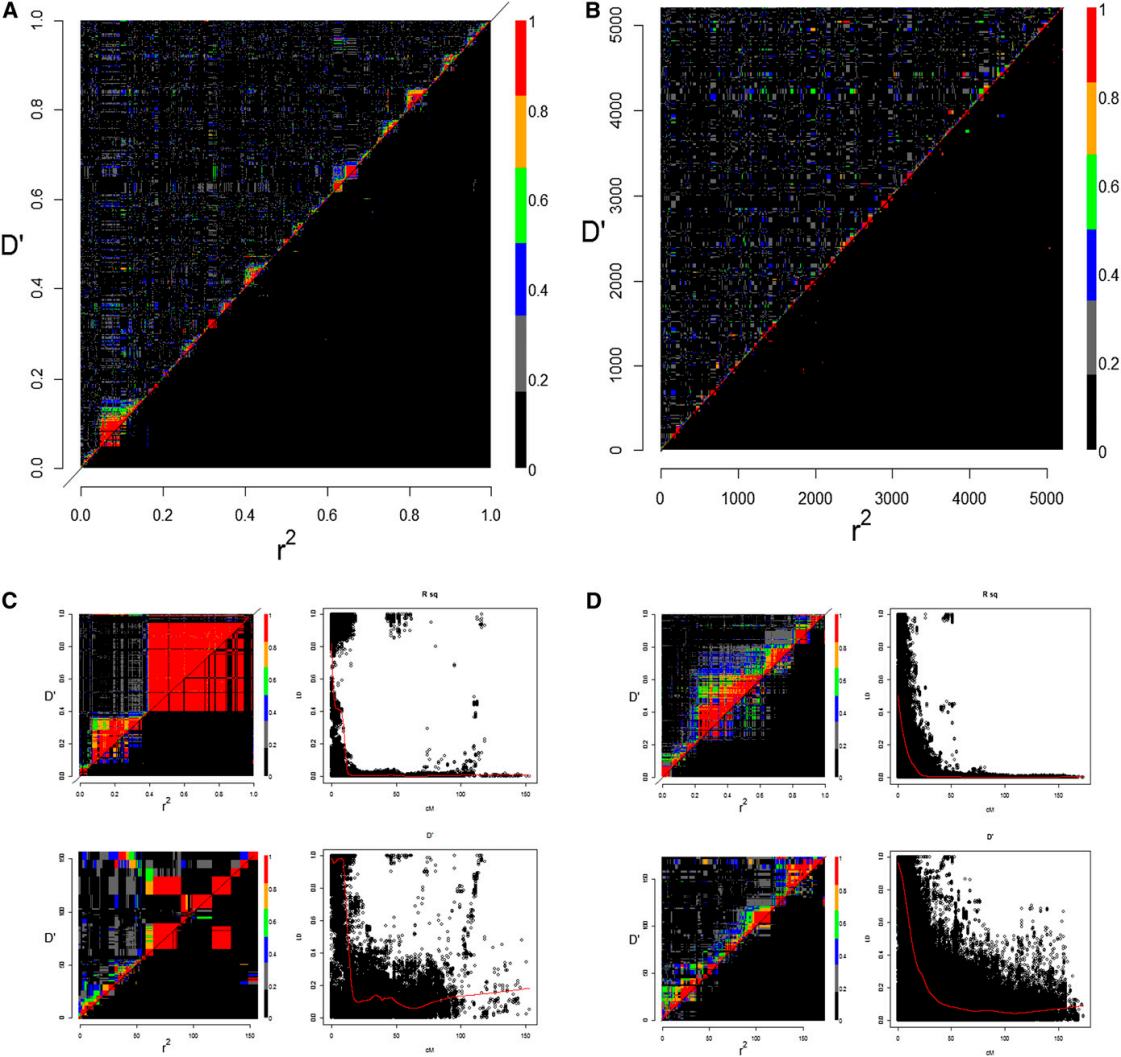
Analysis of LD in the MAGIC population. (A) Heatmap of LD decay of ordered SNPs over 21 chromosomes, ordered 1A, 1B…7D, D′ above the diagonal and r^2^ below. (B) Heatmap of SNPs scaled by the genetic map. Each chromosome has been extended to a nominal 250 cM. Examples of individual chromosomes, LD heatmaps, and plots of LD decay are illustrated for (C) chromosome 2B and (D) chromosome 2D. (C and D) Upper left: ordered SNPs; lower left: scaled by genetic map; upper right: D′ plotted against genetic distance; lower right: r^2^ plotted against genetic distance.

Genotyping 720 of the MAGIC lines and eight founders returned successful marker scoring for 62,543 SNPs. LD decay in the MAGIC population, over the whole genome and chromosome by chromosome, was estimated using 13,074 SNPs segregating in MAGIC for which genetic map positions were available (Wang *et al.* 2014). Heat maps of D′ and r^2^ for ordered markers (Figure 1A) and scaled by genetic distance (Figure 1B) show a clean pattern of LD decay in the MAGIC population. Clusters of high LD among closely linked markers in the analysis of ordered SNPs were not visible when marker order was scaled by genetic map position, illustrating the clustering of SNPs over the genome. Further investigation of LD chromosome by chromosome shows similar patterns, with LD (r^2^) decaying rapidly and cleanly (Figure 1, C and D; Figure S1). The pattern of decay for D′ is also clean, with less long-range LD than usually seen. Exceptions to these simple patterns are most predominant on the D group chromosomes and are likely to result from mapping errors.

### Population validation

Awn presence/absence segregated in the genotyped population with a ratio of 643 (awned):67 (awnless). The observed ratio of 9.60:1 is in agreement with the 7.98:1 ratio expected for a single recessive gene with a frequency of 0.125 after three generations of selfing from a previously random mated population (chi-sq 1df *p*-value 0.15), consistent with genetic control by a single recessive gene.

Seven hundred and ten lines and 8920 SNPs were selected for downstream genetic analysis. The SNPs were selected on genotyping success rate (>0.91) and minor allele frequency (>0.05), with one marker from every pair that correlated perfectly also removed. Any line with marker success rate <75% was deleted.

**Figure 2:**
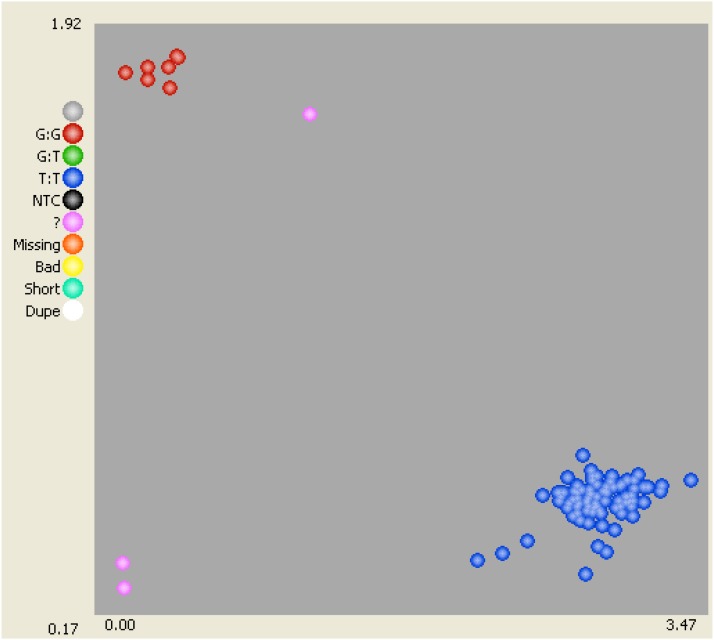
Diagnostic awn presence/absence KASP marker, BobWhite_c8266_227_TG_5AL, genotyped in the AM panel.

Using a Bonferroni corrected 0.01 significance threshold of −log_10_(*p*-value) = 5.95, association of phenotype with all genetic markers found 31 significant SNPs. Of these, 25 had map positions, all on chromosome 5A between 123.8 and 148.3 cM. The peak marker was BobWhite_c8266_227 (−log_10_(*p*-value) = 163.2, chromosome 5AL 140.59 cM). The second highest association was wsnp_Ex_c20899_30011827 (−log_10_(*p*-value) = 111.8, 5AL 147.26 cM) (Table 2). Chromosome arm location was confirmed by BLASTn analysis of flow-sorted wheat chromosome arm sequence data (Table 2). Previous studies show *B1* to be located on chromosome 5A, distal to the chromosomal 4AL/5AL breakpoint (Kato *et al.* 1998). BLASTn analysis of the rice genome identifies the putative ortholog of the most significant SNP identified by mapping (BobWhite_c8266_227) to be LOC_Os03g01170.1 (5.1*e*−111), located within a region previously shown to be collinear with the distal end of cereal group 4 chromosomes (Cockram *et al.* 2010a), and thus involved in the 4AL/5AL translocation. Collectively, these results indicate that mapping in the MAGIC population has detected the *B1* awning locus. Allelic variation at SNP BobWhite_c8266_227 predicted awn phenotype in the MAGIC population for all but 25 lines (3.5%), with C:C and A:A/A:C genotypes highly diagnostic for awns presence or absence lines, respectively (Table 3). Within the 90k SNP array design, this marker assays for polymorphism on the reverse strand of the gene. Analysis of the predicted gene structure in the correct orientation indicated the SNP (G/T) is located in exon 1 at +18 bp relative to the start codon. The SNP was converted to the KASP genotyping platform (LGC Genomics), for independent validation of the 5AL awning locus identified in MAGIC, genotyped on a wheat AM panel consisting of 376 varieties genotyped with 1621 DArT and phenotyped for awn presence/absence (Figure 2; primer sets listed in Table S2). Subsequent genome-wide association analysis in the AM panel identified three significant [−log_10_(*p*-value) > 5.21] markers: wPt_6462 (the last mapped marker on chromosome 5AL, 123.91 cM (Huang *et al.* 2012), BobWhite_C8266_227_TG_5AL (5AL, 140.59 cM in the 90k SNP consensus map) (Wang *et al.* 2014), and one unmapped marker, allocated to pseudochromosome 22 (BWS5497_CT). Of these, the KASP marker tagging the 5AL genetic locus first identified in the MAGIC population was by far the most significant [−log_10_(*p*-value) = 36.42]. Of the 337 AM varieties possessing both genotype and phenotype data, allelic calls at KASP marker BobWhite_c8266_227_TG_5AL predicted awned (G:G genotype) and awnless (T:T) phenotype in all but three instances (Table S1). Subsequent QTL analysis of the AM population using the peak marker as a covariate identified no additional significant associations.

**Table 2:** Details of SNPs significantly associated with awn presence/absence in the MAGIC population

SNP Name	SNP	Brachypodium	Rice	Sorghum	Wheat GSS Hit	Wheat Chromosome*^a^*
BobWhite_c8266_227	A/C	Bradi1g78400	Os03g01170	Sb01g050580	5AL_2805500	5AL
wsnp_Ex_c20899_30011827	T/C	Bradi1g77770	Os3g01250	Sb01g049780	5AL_2699187	5AL

Putative orthologs in the sequenced genomes of Brachypodium, rice, and sorghum are listed. Sequence contigs identified from searches of wheat genome survey sequence (GSS) generated from flow-sorted chromosome arms are indicated.

a Genetic map location based on the 90k consensus map (Wang *et al.* 2014).

**Table 3:** Allele frequencies at SNP marker BobWhite_c8266_227 for awns presence/absence phenotypic classes in the 710 MAGIC lines analyzed

	A:A	A:C	C:C
Absence	625	3	15
Presence	8	2	57

The allele associated with presence of awns at BobWhite_c8266_227 is carried by the founder variety Soissons and no other founder. Soissons is also the only awned founder line. Forty-six mapped SNPs on chromosome 5A were unique to Soissons among the founders. Of these, those immediately flanking BobWhite_c8266_227 are strongly associated with awns [wsnp_Ex_c20899_30011827 at 147.26 cM, −log_10_(*p*-value) = 111.8 and RAC875_c61559_435 at 139.76 cM, −log_10_(*p*-value) = 139.8]. The large reduction in −log_10_(*p*-value) from the peak to the flanking markers can be used to define an interval of 7.5 cM to contain the QTL with high confidence. Two other markers also lie within this interval on the consensus map. First, wsnp_Ex_c16018_24438963 shows no association with awning but correlates highly with markers on other chromosomes and is most likely incorrectly mapped or otherwise problematic. RAC875_c8642_231 correlates perfectly with BobWhite_c8266_227 (and was therefore not analyzed for association with awning) but is mapped to 141.7 cM, adjacent to the peak marker. The rapid decline in strength of association with map distance, together with the closeness of the centromeric in comparison with the teleomeric flanking marker, indicate that higher SNP density in this region may improve precision.

To investigate the predicted gene content of the wheat *B1* region, comparative analysis with rice was undertaken. The rice orthologs of BobWhite_c8266_227 and the second peak SNP are LOC_Os03g01170 and LOC_Os03g01250, respectively. This interval in rice spans ∼50 kb on chromosome Os03 and contains 10 genes. Marker RAC875_c8642_231, removed from the scan due to perfect genotypic correlation with the peak marker, was found to originate from the same gene and so did not further help collinear analyses. The rice gene, *An-1*, which controls awning in rice (Luo *et al.* 2013), is not in the 50-kb interval. BLASTn analysis of the rice genome with *An-1* CDS identifies five significantly similar rice genes (<1e−10), none of which are on Os03. No obvious candidate genes were identified within the 50-kb region in rice.

### Phenotype data

With the exception of awns, all traits and SNP data have been modeled simultaneously with the SNP data using Bayesian Networks. Results are reported and discussed by Scutari *et al.* (2014). Trait means and SNP data for the MAGIC lines are available at http://www.niab.com/MAGIC/.

## Discussion

### The timeliness of MAGIC

Much of the potential for fine-mapping in crops has remained largely unrealized, because progress in mapping population design has failed to match rapid advances in genotyping technologies. The recent advent of plant MAGIC populations has the potential to address this disparity. By combining controlled allelic inputs from multiple parents with high levels of genetic recombination achieved over multiple rounds of intercrossing, MAGIC populations overcome the specific drawbacks associated with traditional bi-parental populations, as well as more recent AM approaches (Mackay and Powell 2007). The higher allelic diversity in MAGIC improves sampling of available genetic diversity and facilitates the analysis of interacting or complex traits within a unified mapping population. These factors make MAGIC populations ideal platforms for high-resolution genetic dissection of QTL, the anchoring of physical maps, and as community-based resources for crop improvement. Such activities will be further enhanced by the application of emerging high-throughput phenomics platforms (Gegas *et al.* 2014). However, MAGIC population creation takes more time and greater effort than for other artificially constructed populations. We initiated this project in 2006, reasoning at the time that improved genomic resources would soon become available in wheat. This has been proven correct: high-density panels of SNPs are increasingly available in wheat (Paux *et al.* 2012), with recent advances increasing numbers from 9000 in 2012 (Cavanagh *et al.* 2013) to 90,000 in 2013 (Wang *et al.* 2014) and 820,000 in 2014 (http://www.cerealsdb.uk.net).

### MAGIC population properties

This wheat MAGIC population was created with founders selected as important for breeders’ current priorities. This strategy has been justified: the MAGIC population is more diverse than the UK reference Avalon × Cadenza population but less diverse than the panel of 64 varieties used here for comparison. This is perhaps not surprising given the number of founders involved, but it confirms one objective of multi-founder populations: to capture more genetic diversity. A disadvantage of AM is that changes in allele frequencies controlling targeted traits occur over time due to breeder selection, and the trend of close kinship between varieties of similar release year (White *et al.* 2008), can limit power to detect QTL. This is due to the need to correct for kinship: on adjustment, loci tagging QTL whose frequencies have changed will have their power of detection reduced. QTL of large effect will have changed most in frequency over time and will be most affected by the loss of power, whereas QTL that have changed little in frequency are likely to be of smaller effect and may be undetectable. Use of experimental populations such as MAGIC avoids this. However, MAGIC and AM approaches can be complementary: where close genetic relationships between the two are present, each is a potential replication set for the other. In human genetics, such replication studies are treated as of great importance (Chanock *et al.* 2007), attention to which is now being made in plants (Hall *et al.* 2010).

Over the whole genome, the pattern of LD decay in the MAGIC population is clean. Within chromosomes, patterns vary from chromosome to chromosome. The vertical bands of high r^2^ found on some of the plots (*e.g.*, 5A and 7A) probably arise from errors ordering markers in the consensus map and are likely a result of relatively low population size and map amalgamation between the constituent mapping populations, resulting in inaccuracies.

### Genetic control of awning

The presence of awns has been associated with, or tightly linked to, numerous beneficial traits (Bariana *et al.* 2006). The *B1* awning locus has previously only been coarsely mapped within genetic intervals flanked by restriction fragment length polymorphism (RFLP) markers [57.3 cM and 20.4 cM intervals by Kato *et al.* (1998) and Bariana *et al.* (2006), respectively]. Here, we map *B1* to a 7.5-cM interval and identify a highly diagnostic marker for the *B1* awning locus that we validate in an independent AM panel. The availability of diagnostic markers in cereals (Cockram *et al.* 2009, 2010b, 2012) facilitates efficient tracking and manipulation of alleles and allele combinations. Conversion of the highly diagnostic marker for the *B1* locus identified in the SNP array to the KASP genotyping platform will allow flexible deployment by wheat researchers and breeders. However, the association of marker BobWhite_c8266_227 and phenotype in both the MAGIC and AM populations was not perfect, and could be due to one of the following factors:

(i) Segregation at other loci. Allelic combinations at the *HD*, *B1*, and *B2* loci and their effects on phenotype have previously been described (Watkins and Ellerton 1940; McIntosh *et al.* 1998), indicating that the awned MAGIC founder Soissons possess homozygous recessive *b1*, *b2*, and *hd* alleles. The remaining seven MAGIC founders are all awnless and are predicted to carry dominant inhibitors at two of the three awning loci: *B1* and *B2* or *B2* and *Hd*. Genetic analysis in the MAGIC population identified significant markers only for the *B1* locus, whereas phenotypic analysis did not identify any additional awn phenotypes such as bearded (*b1b1,b2b2,hdhd*), tipped (*B1B1,b2b2,hdhd*), hooded (*b1b1,b2b2,HdHd*), or hooded beardless (*B1B1,B2B2,HdHd*). These results indicate segregation only at the *B1* locus in the MAGIC population, and that all seven awnless founders carry the *B1B1,B2B2,hdhd* haplotype. Similarly, *B1* was the only known awning locus identified in the AM panel, indicating that the bsence of awns in the germplasm investigated is predominantly conferred by the *b1b1,B2B2,HdHd* haplotype. The failure to detect significant associations in either population other than at the *B1* locus indicates no additional modifiers of awning were segregating. Collectively, these results indicate that imperfect linkage of the peak marker with phenotype was not due to segregation at other awning loci.
(ii) Incomplete linkage between the marker and *B1*. While wheat lacks a sequenced physical map, comparative analyses with the sequence genomes of brachypodium, rice, and sorghum allowed possible gene content and candidate genes to be identified. To date, two genes controlling awning in grass species have been map-based cloned: *Awn-1* (*An-1*) in rice (Luo *et al.* 2013) and *SHORT INTERNODES* (*SHI*) in barley (Yuo *et al.* 2012). However, we show that their rice orthologs are not located within the rice physical region collinear with *B1*. Furthermore, wheat homologs of these genes do not map to the long arm of chromosome 5A. No obvious candidate genes for the control of awning were identified in the 50-kb region of rice defined by colinearity with the two peak markers. However, the peak awn marker is predicted to be approximately five genes away from the *ZCCT* genes underlying the major vernalization response locus *VRN-A^m^2* in wheat (Cockram *et al.* 2010b). Because few genes have been map-based cloned to date, this is an unexpected result, and it would be interesting to investigate further if the preference for awned varieties in warmer climates may be due to effects at *VRN2*.

## Conclusions

The UK wheat MAGIC population described here has taken a long time to develop. However, the composition and size of the population justify the effort. This work demonstrates that MAGIC populations can be deployed to map genetic loci within the complex 17 GB hexaploid wheat genome to small genetic intervals. It also confirms the complementariness of MAGIC and AM populations for QTL cross-validation. The high genetic diversity and genetic recombination captured in comparison with standard bi-parental populations make MAGIC populations ideally suited to exploit current rapid advancements in high-throughput genotyping and phenomics platforms in wheat and other crops. Over time, the layering of phenotypic datasets will allow comparison of QTL actions and interactions simultaneously across multiple traits in MAGIC populations. This process has been started with the analysis of Scutari *et al.* (2014). Such populations and approaches are poised to open new avenues for genetic analysis in plants. Data and instructions for access to seed are available from http://www.niab.com/MAGIC/.

## Supplementary Material

Supporting Information
