# Supplementary material for: An Eight-Parent Multiparent Advanced Generation Inter-Cross Population for Winter-Sown Wheat: Creation, Properties, and Validation
Source: G3 (Bethesda). 2014 Sep 1;4(9):1603–10. doi: 10.1534/g3.114.012963 (PMC4169152; doi:10.1534/g3.114.012963)
Supplement: Supporting Information [file supp_4_9_1603__index.html]

Supporting Information 

# An Eight-Parent Multiparent Advanced Generation Inter-Cross Population for Winter-Sown Wheat: Creation, Properties, and Validation

## Supporting Information for Mackay *et al.*, 2014

**Files in this Data Supplement:**

- Supporting Information - Figure S1 and Tables S1 and S2 (PDF, 141 KB)
- Figure S1 - MAGIC progeny LD heatmaps and LD decay plots for the 21 wheat chromosomes based on 13074 mapped and segregating SNPs (PDF, 842 KB)
- Table S1 - Wheat varieties in the AM panel used to validate the KASP (PDF, 121 KB)
- Table S2 - KASP primers for marker BobWhite\_c8266\_227\_TG\_5AL (PDF, 110 KB)
