# Supplementary material for: An Eight-Parent Multiparent Advanced Generation Inter-Cross Population for Winter-Sown Wheat: Creation, Properties, and Validation
Source: G3 (Bethesda). 2014 Sep 1;4(9):1603–10. doi: 10.1534/g3.114.012963 (PMC4169152; doi:10.1534/g3.114.012963)
Supplement: Supporting Information [file supp_4.9.1603_FigureS1.pdf]

Figure S1 MAGIC progeny LD heatmaps and LD decay plots for the 21 wheat chromosomes based on 13074 mapped and segregating SNPs. For each chromosome: Top left: heatmap of LD among ordered markers. D’ above diagonal, r^2 below. Bottom left: heatmap scaled by genetic map. Top right LD decay (r^2) plotted against genetic distance between marker pairs. Bottom right: LD decay (D’) plotted against genetic distance between marker pairs.

All, no map

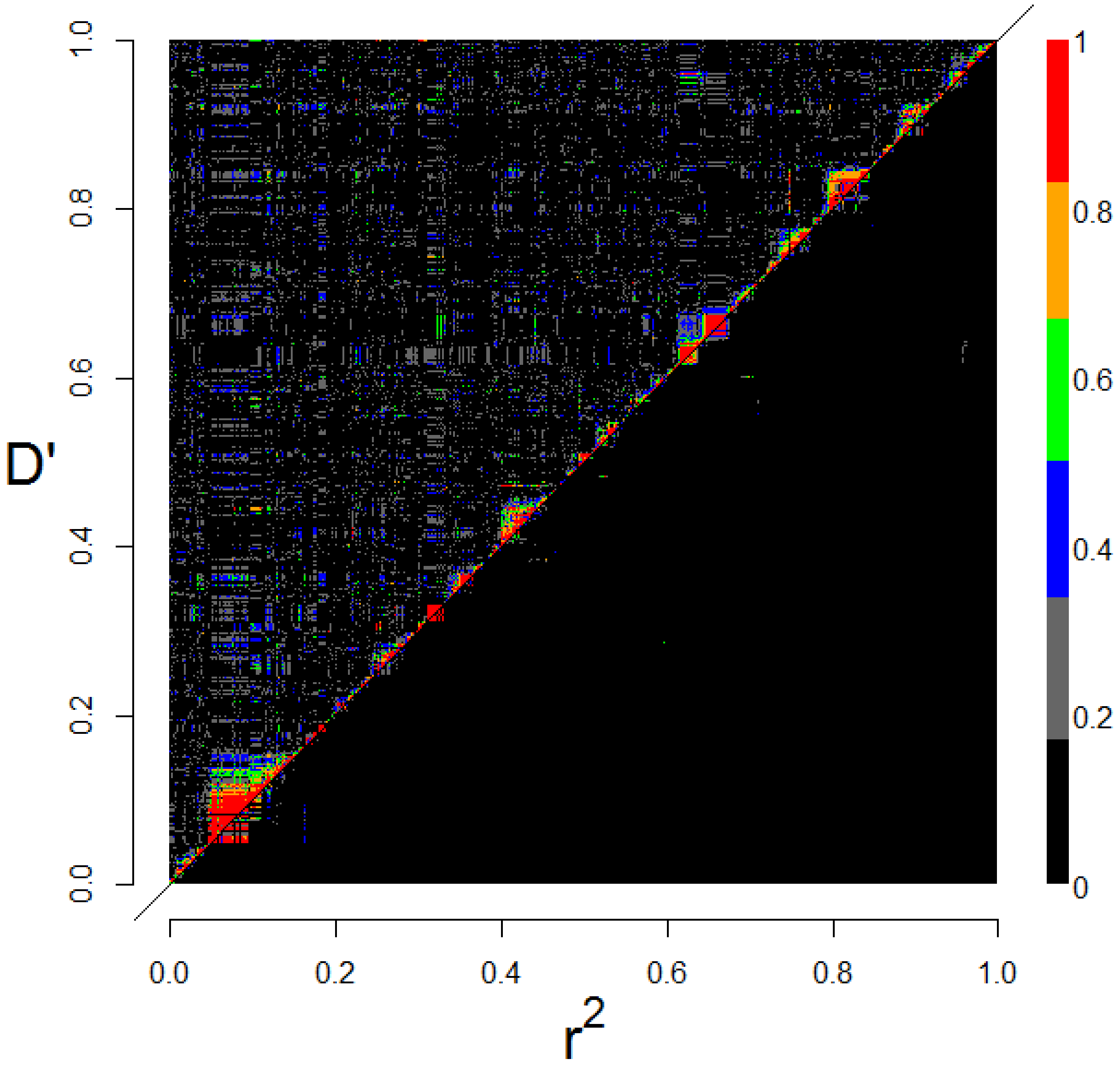

All, with map, change chromosome every 250 cM

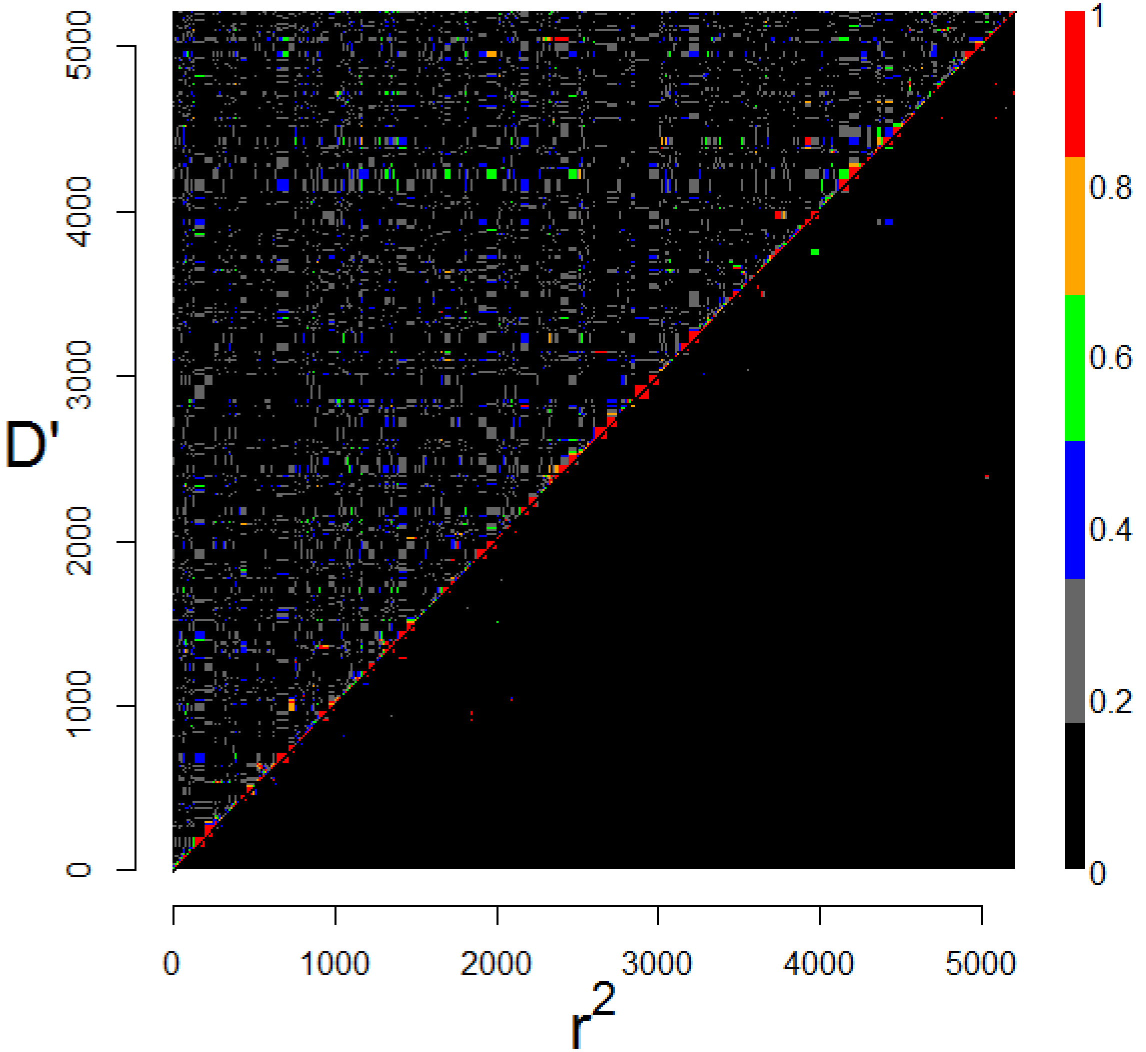

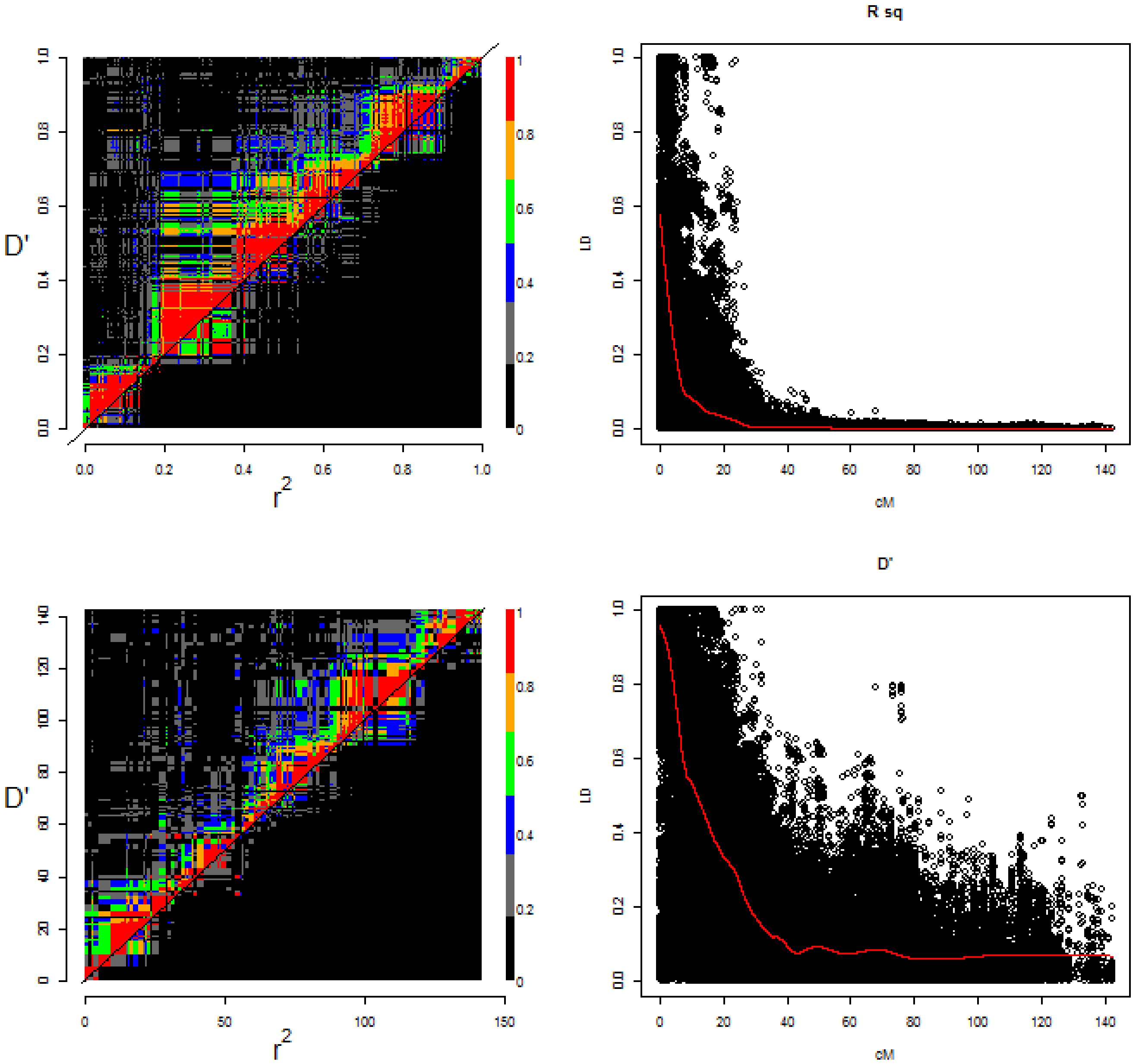

1A

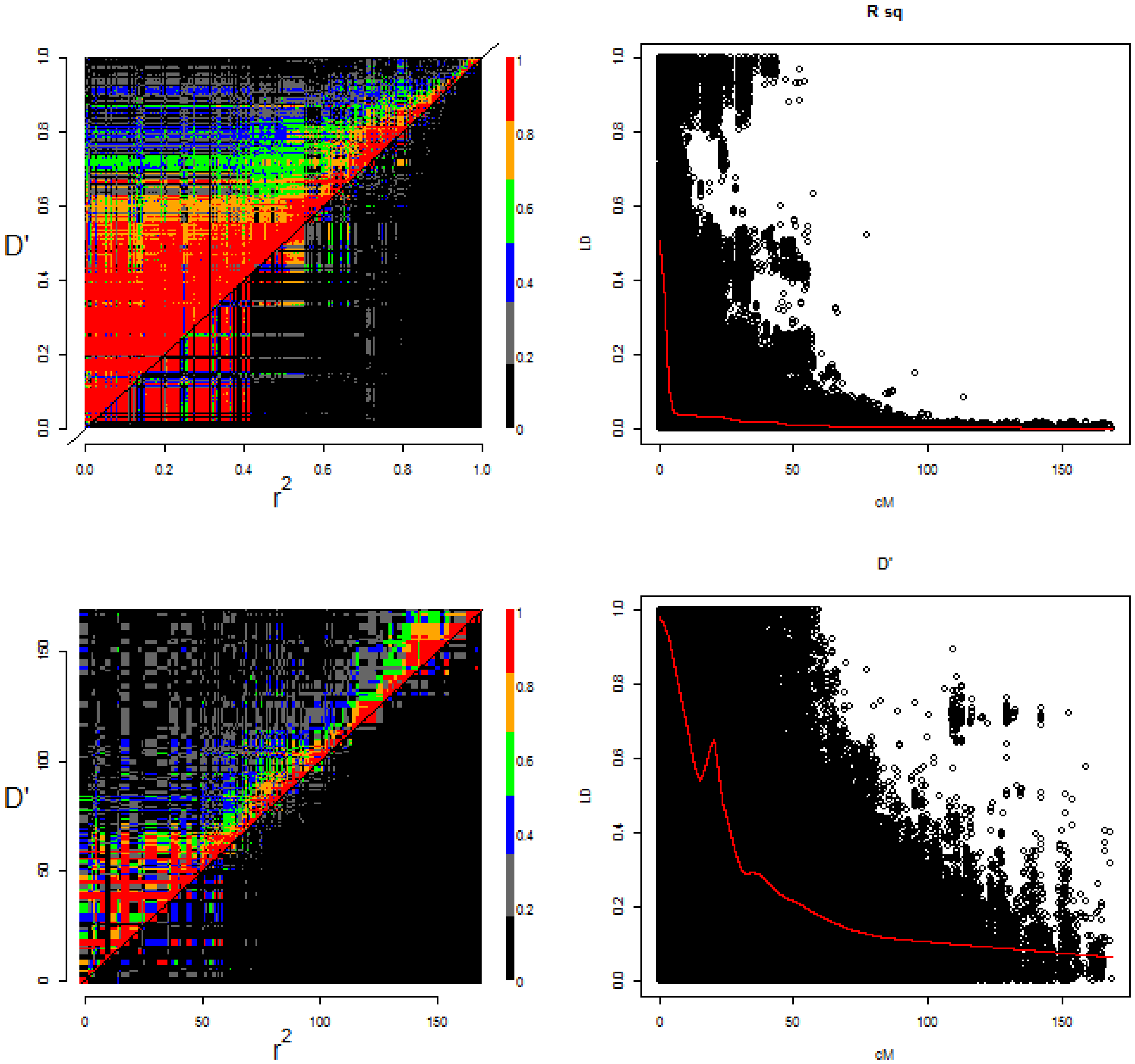

1B

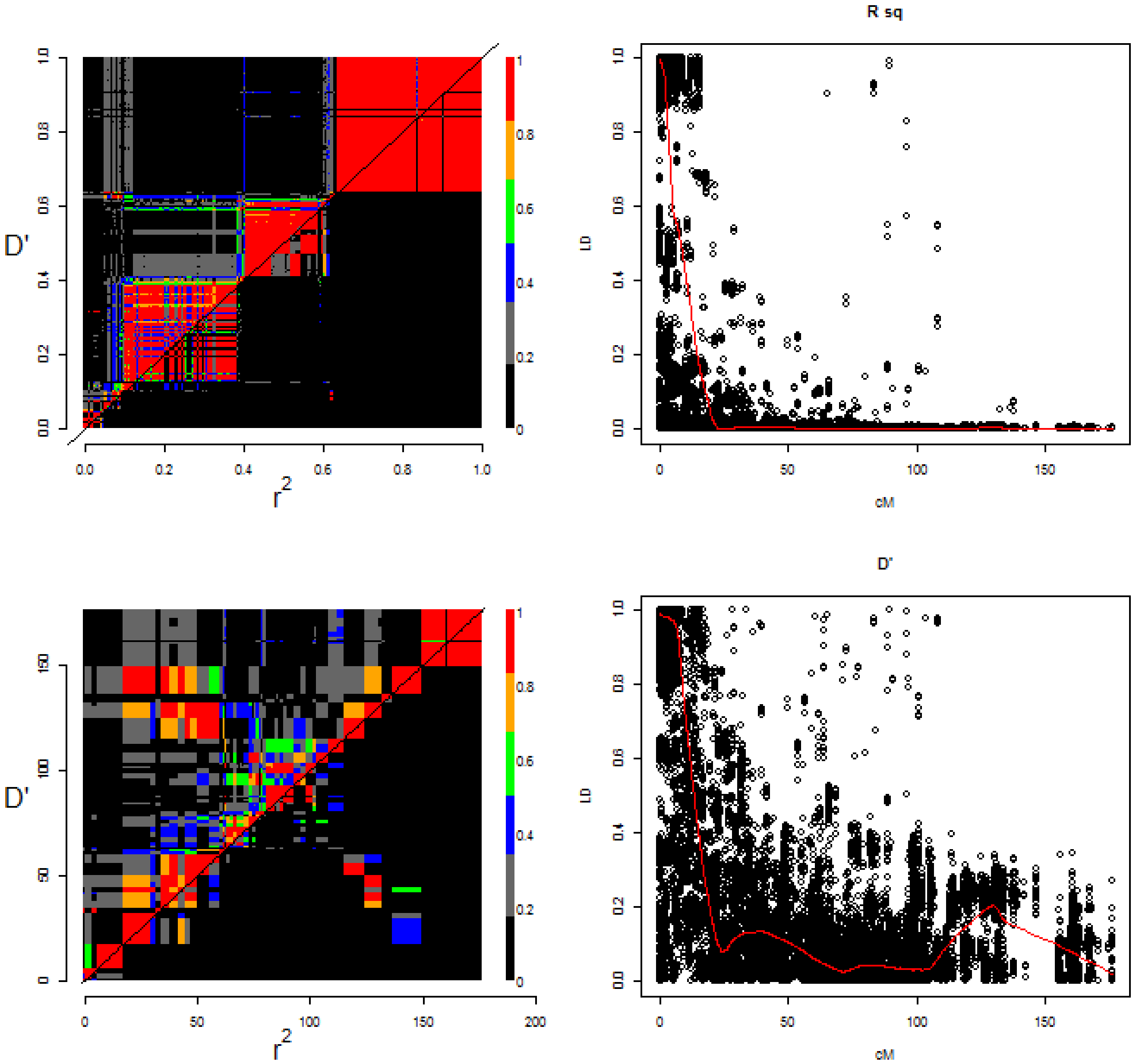

1D

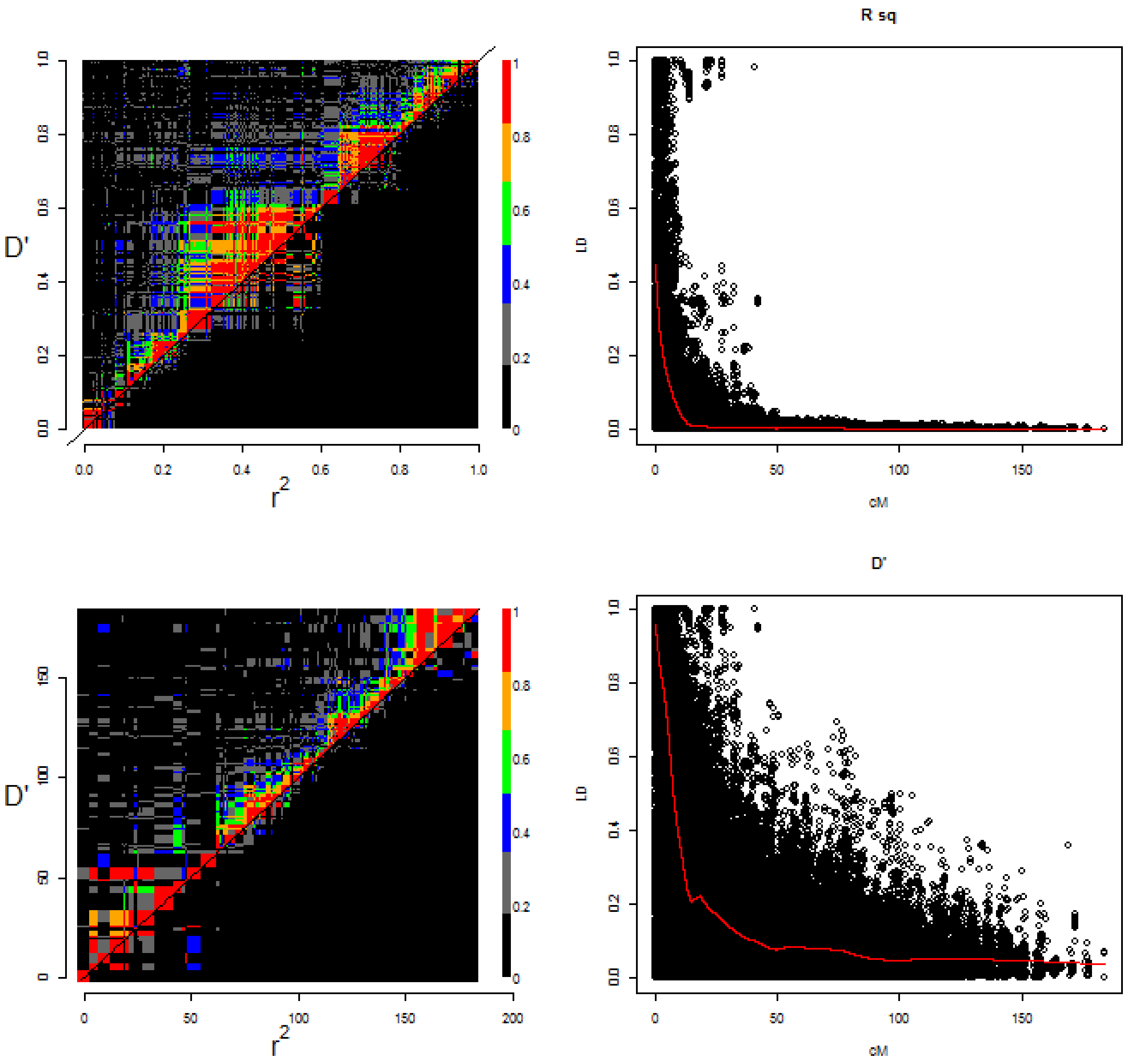

2A

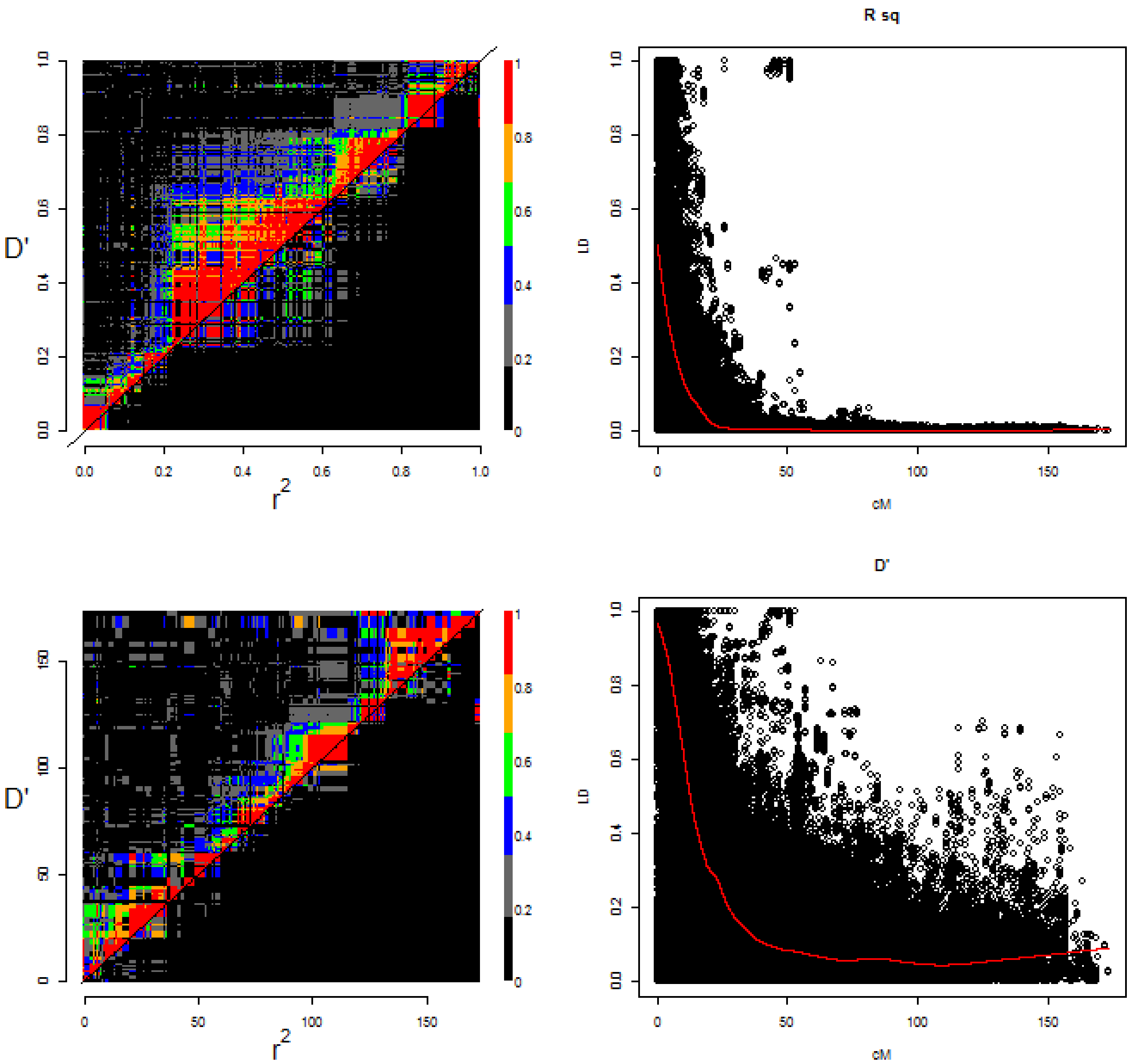

2B

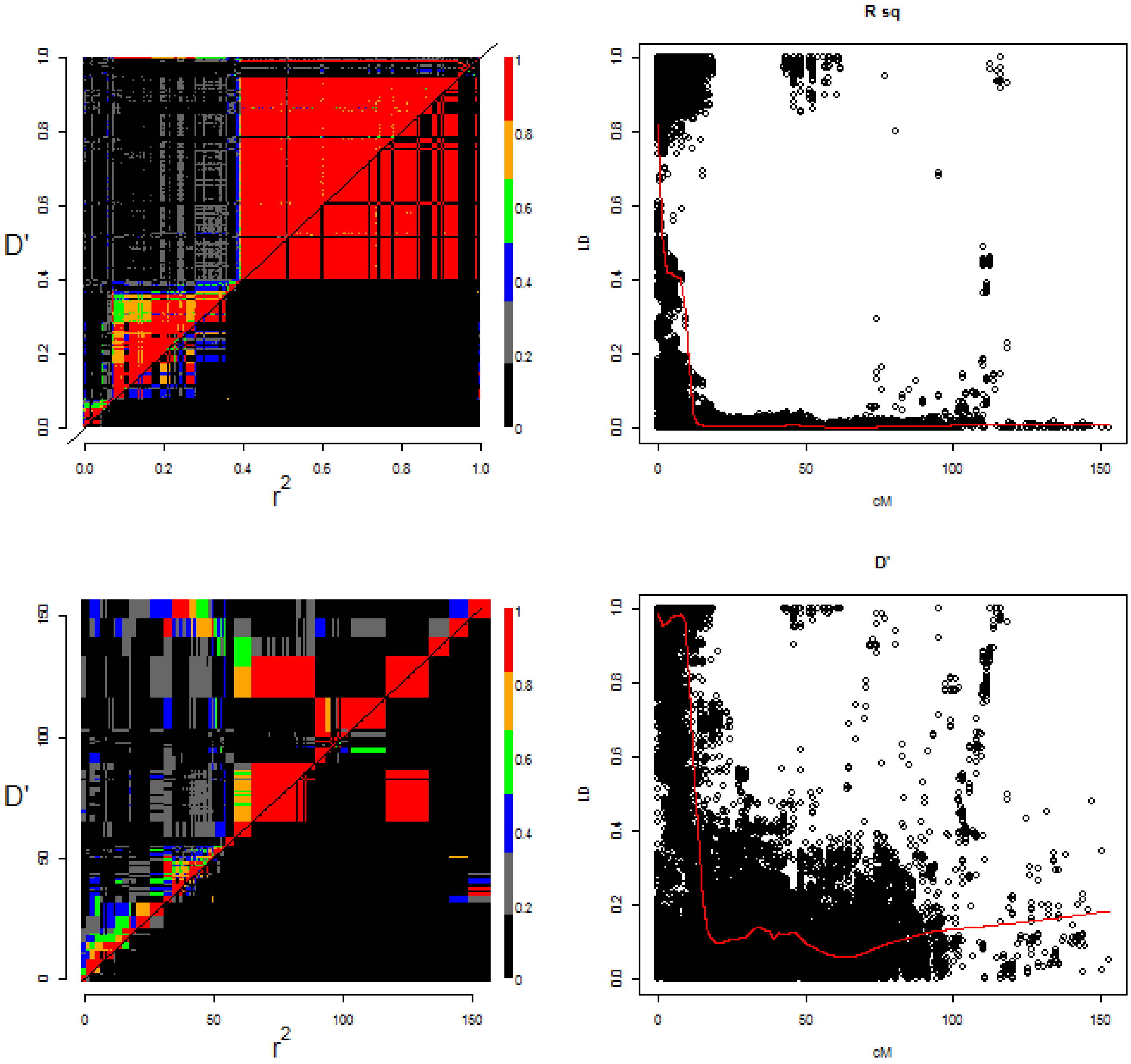

2D

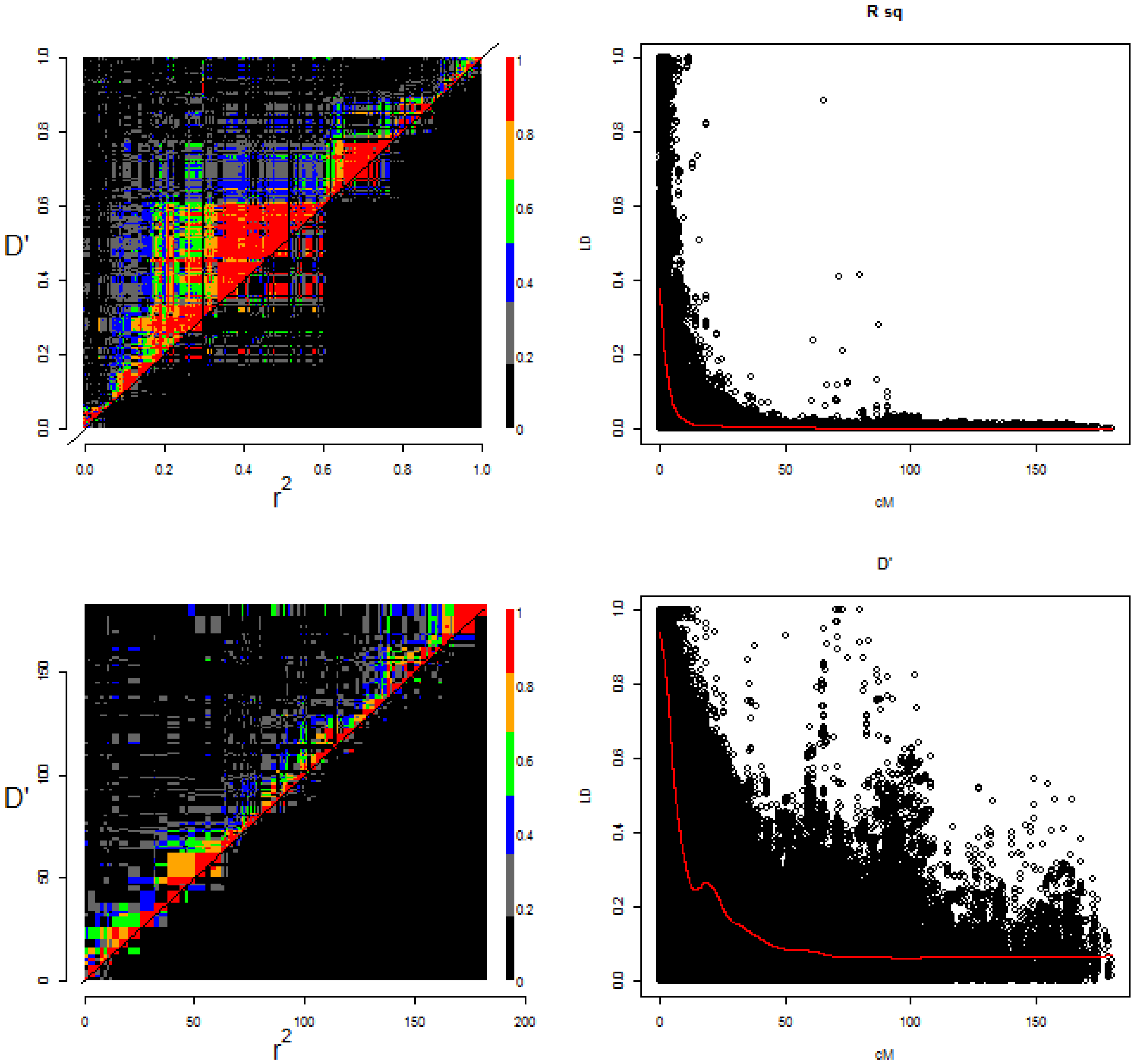

3A

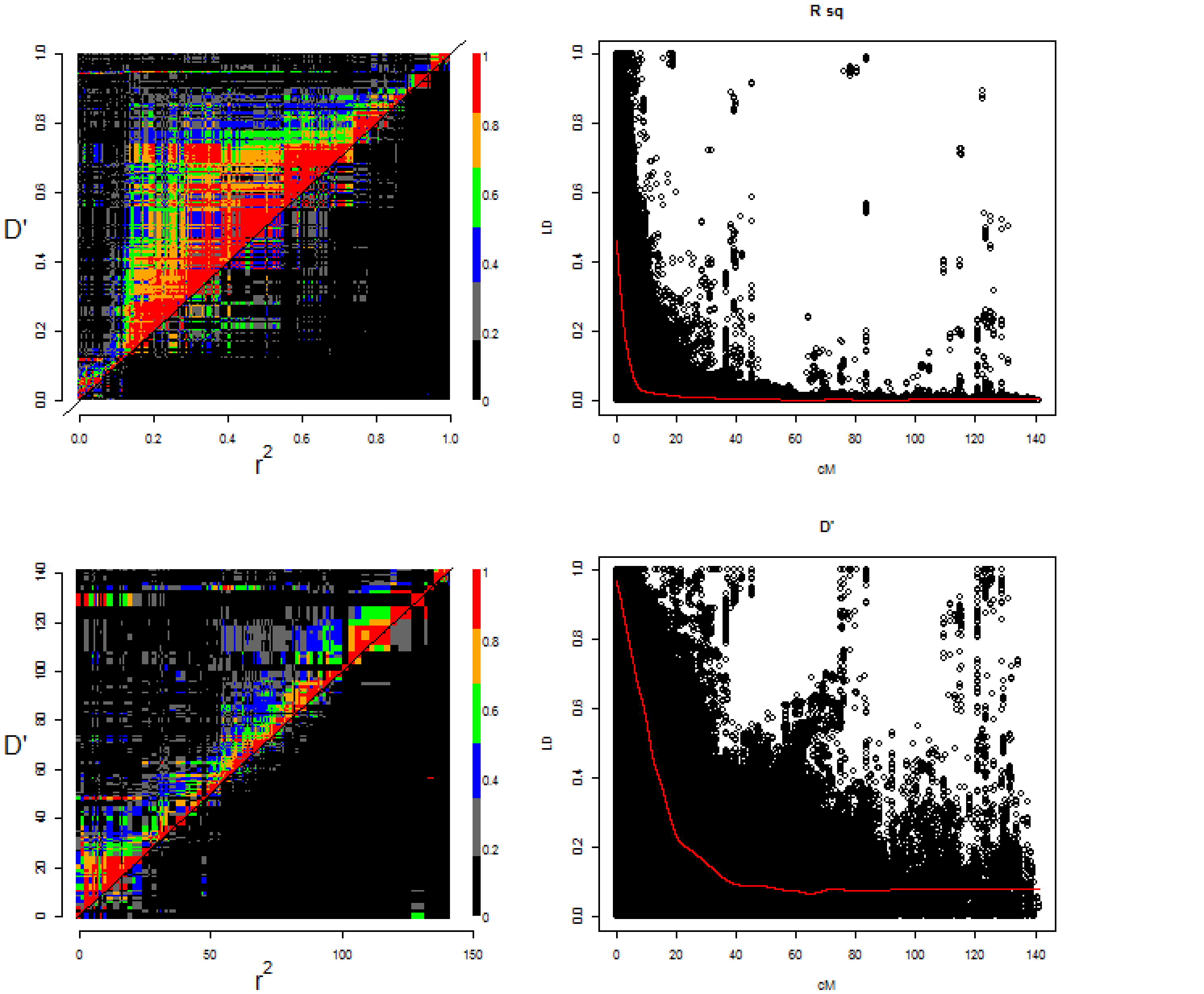

3B

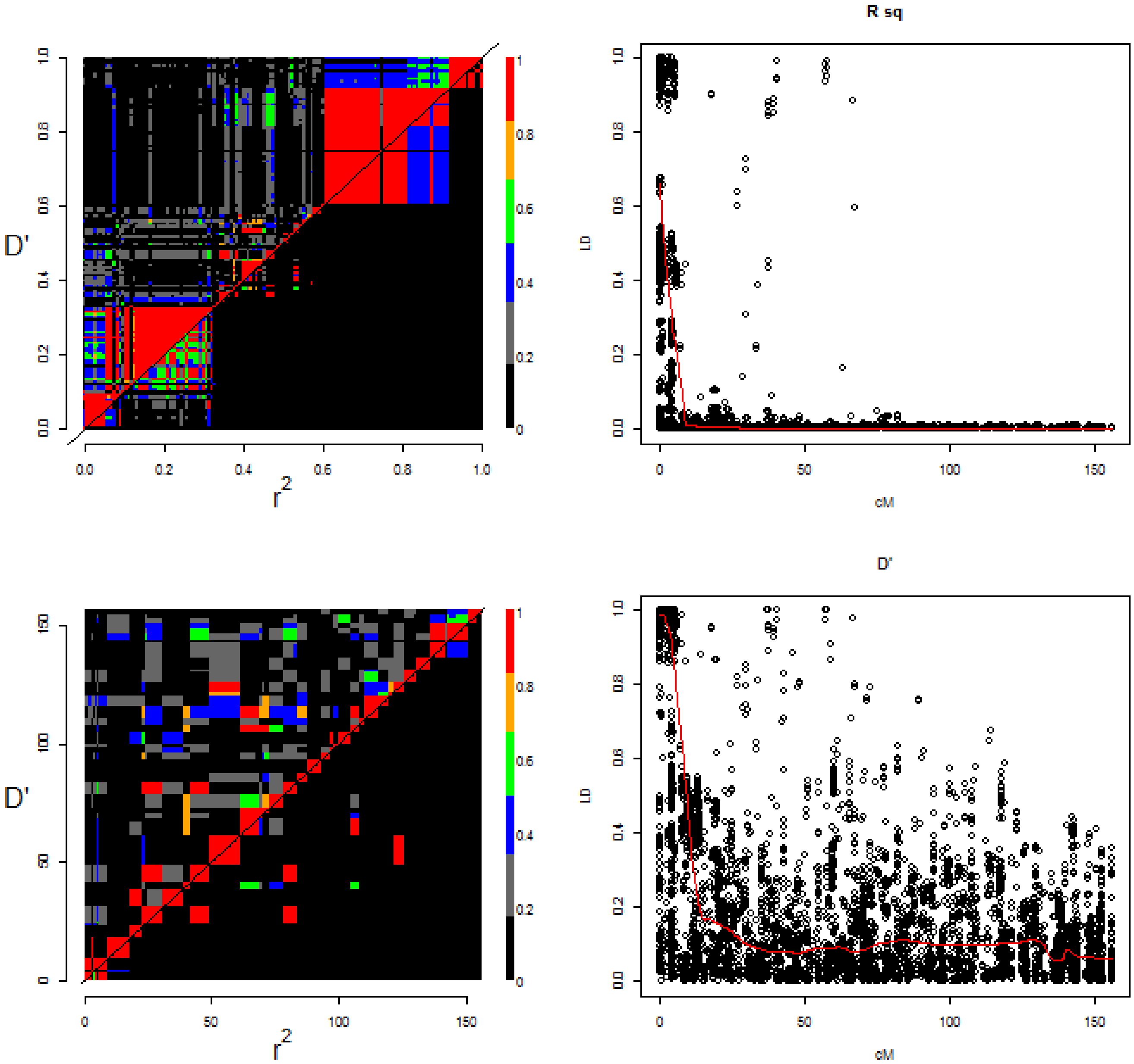

3D

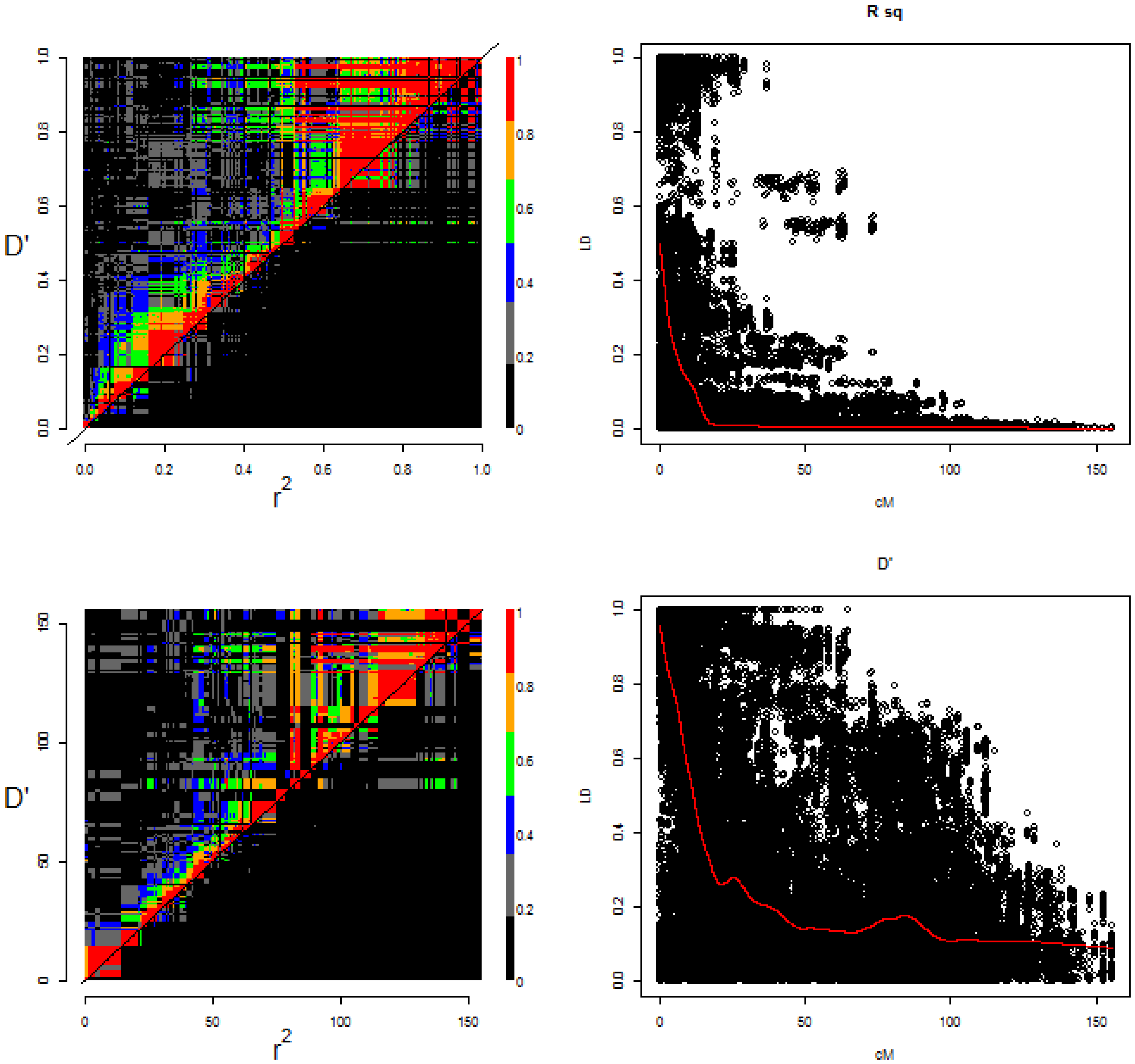

4A

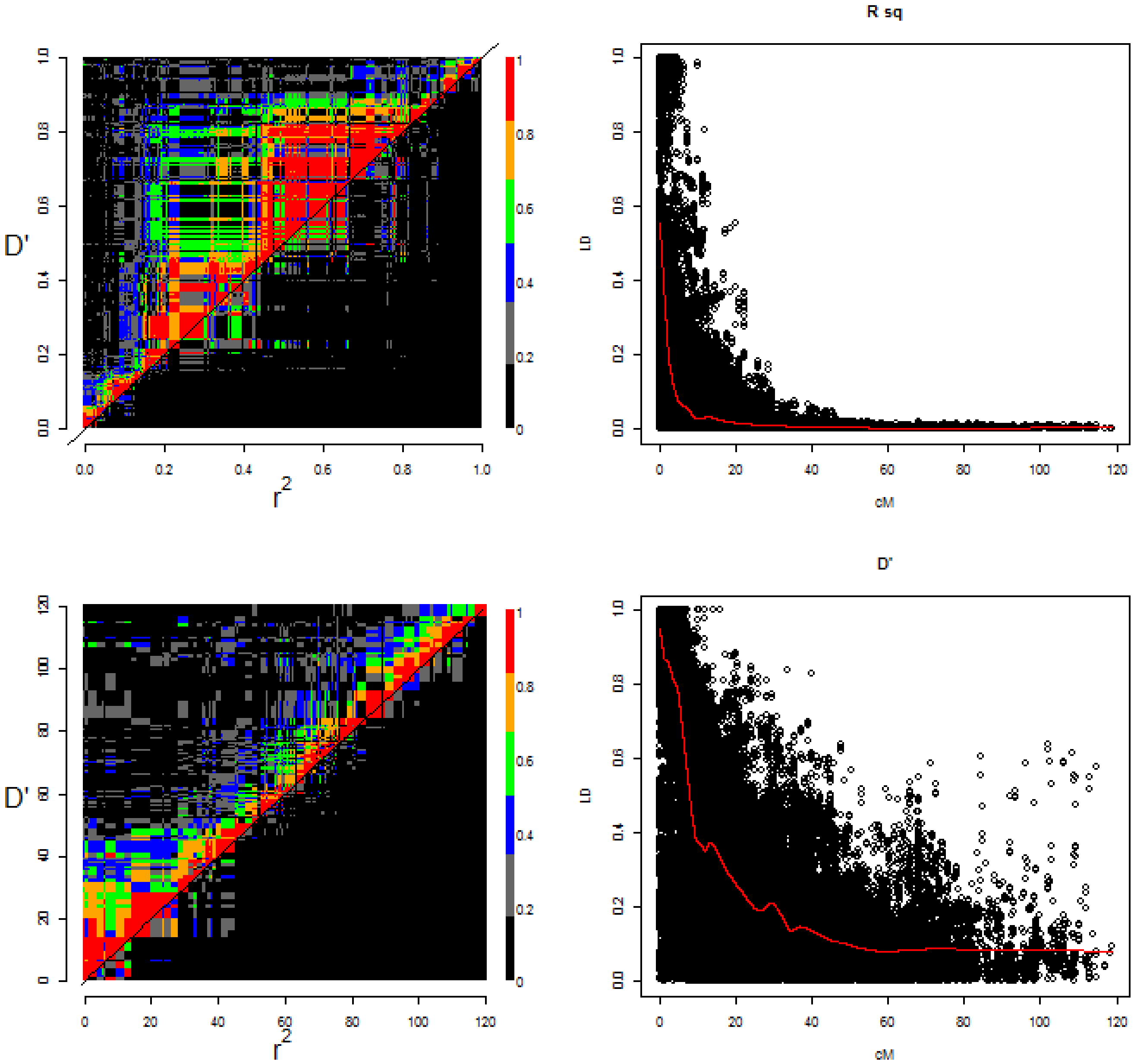

4B

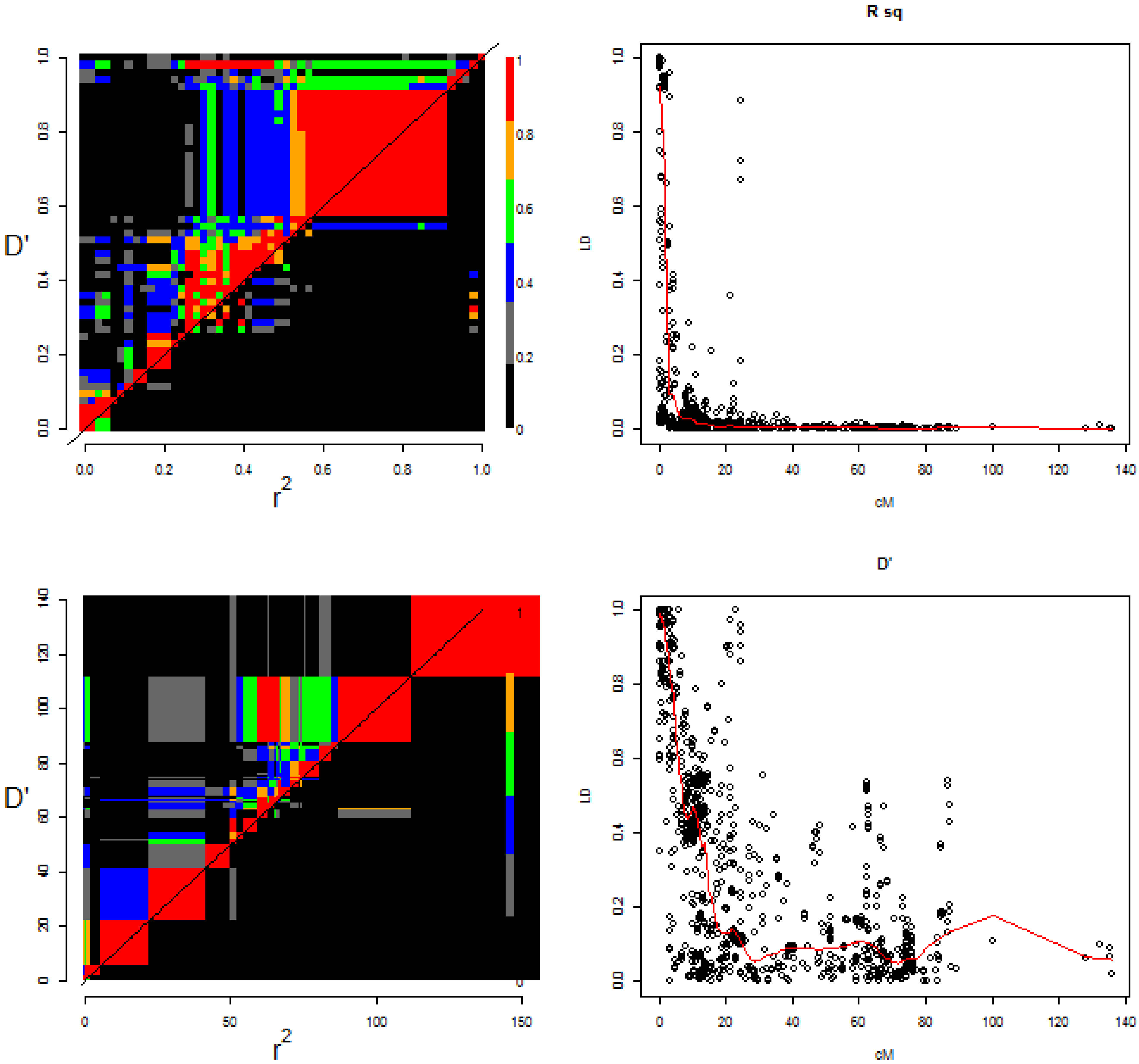

4D

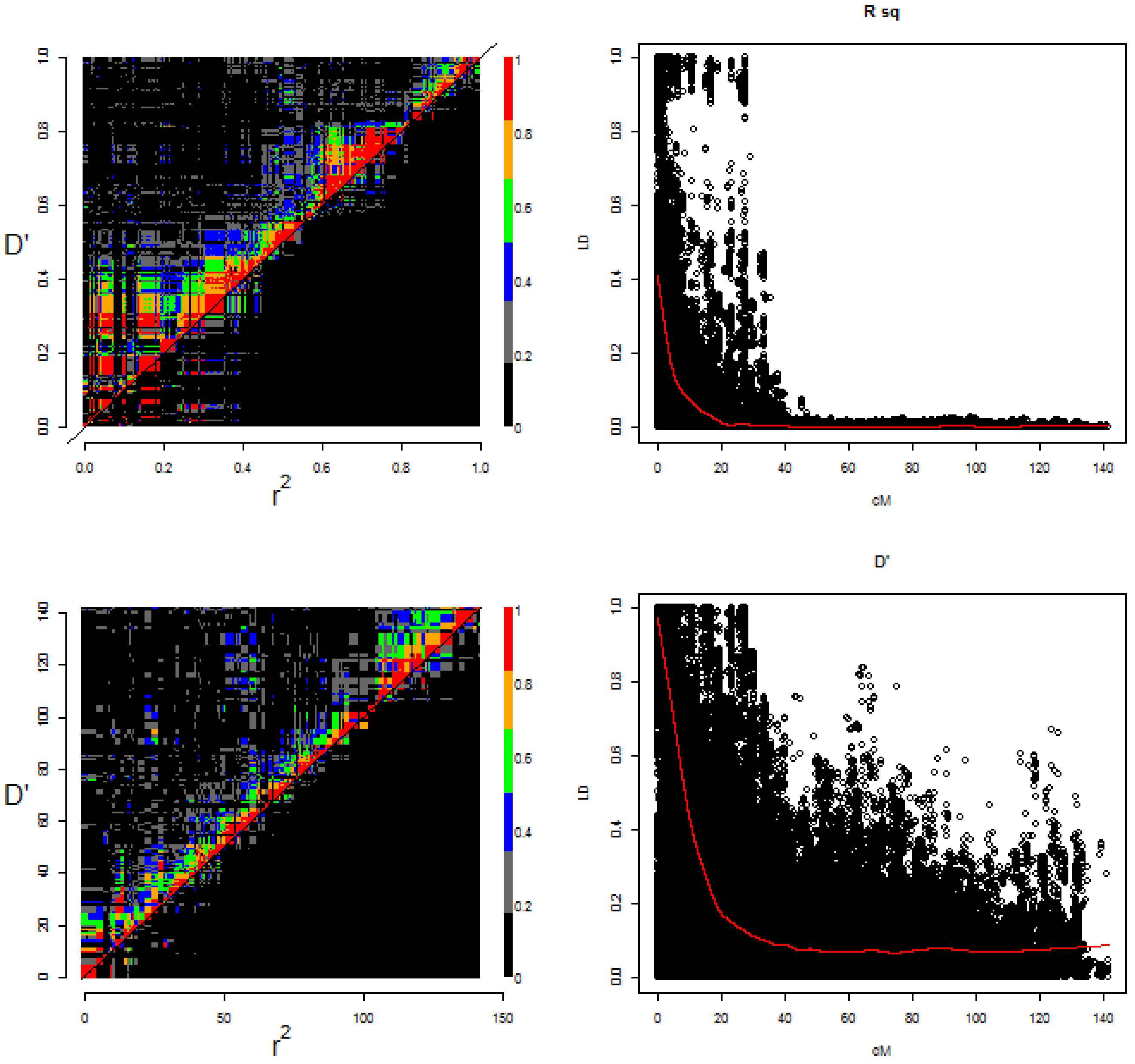

5A

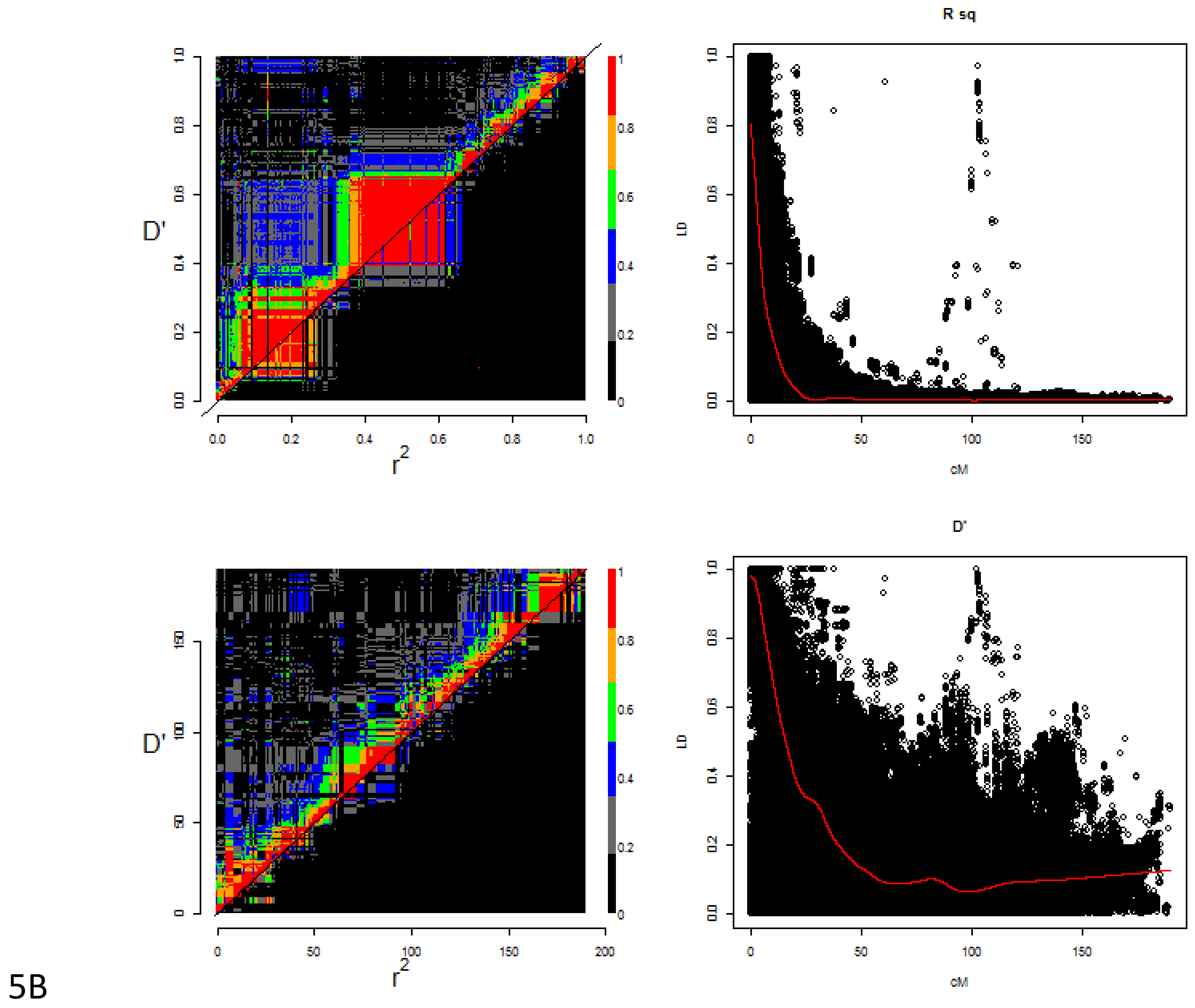
R sq
D'
LD
cM
D'
r2
5B

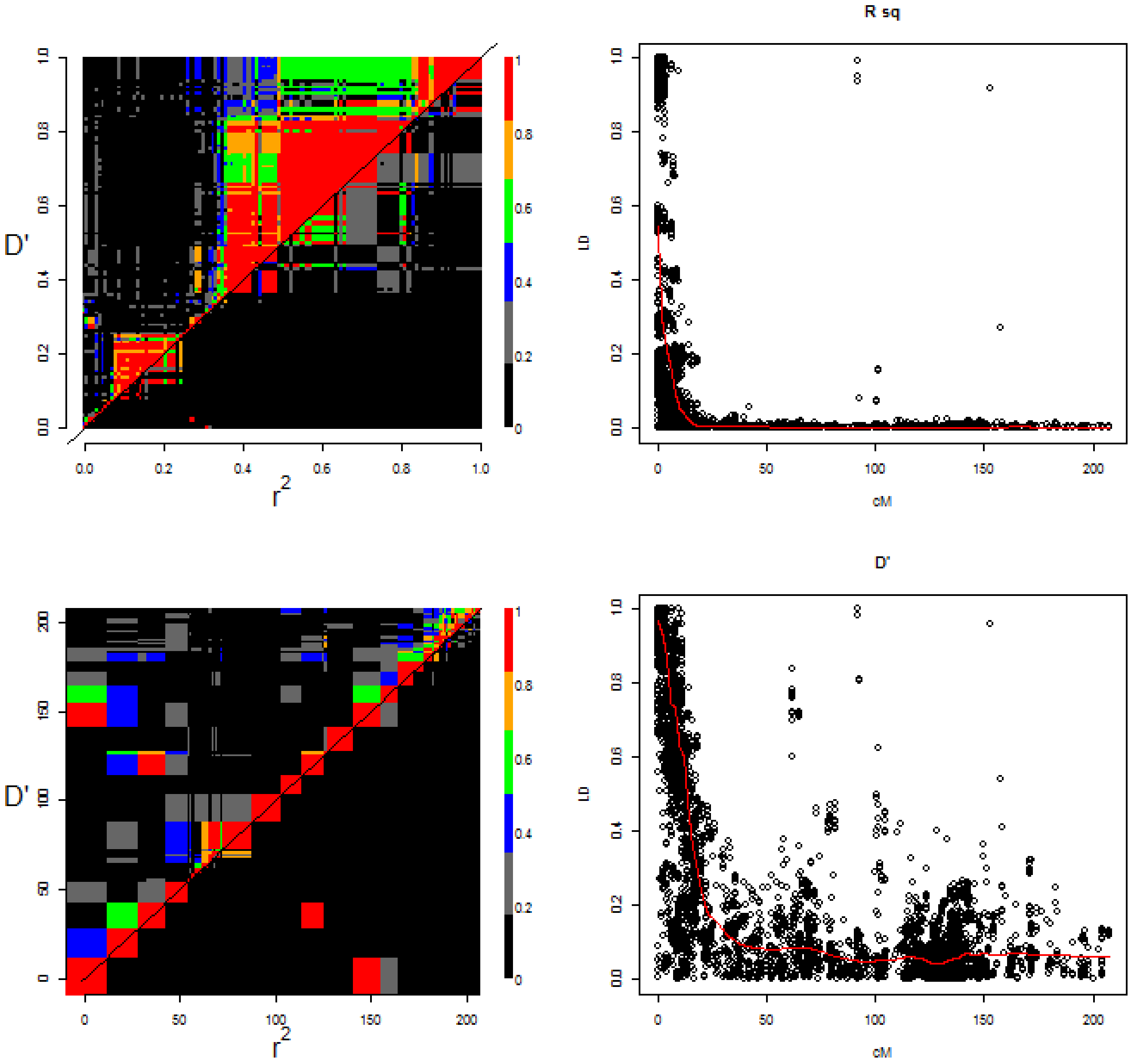

5D

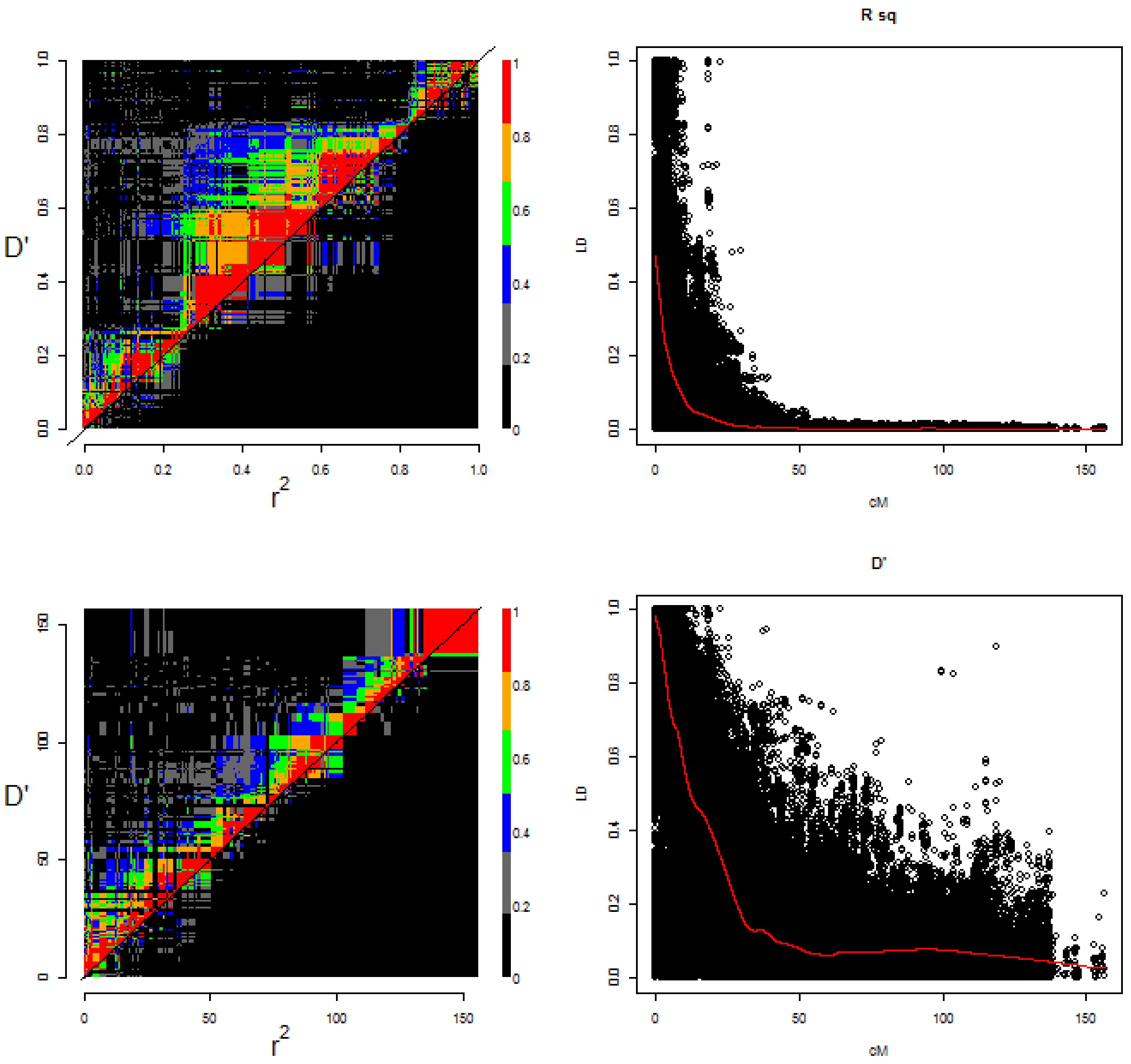

6A

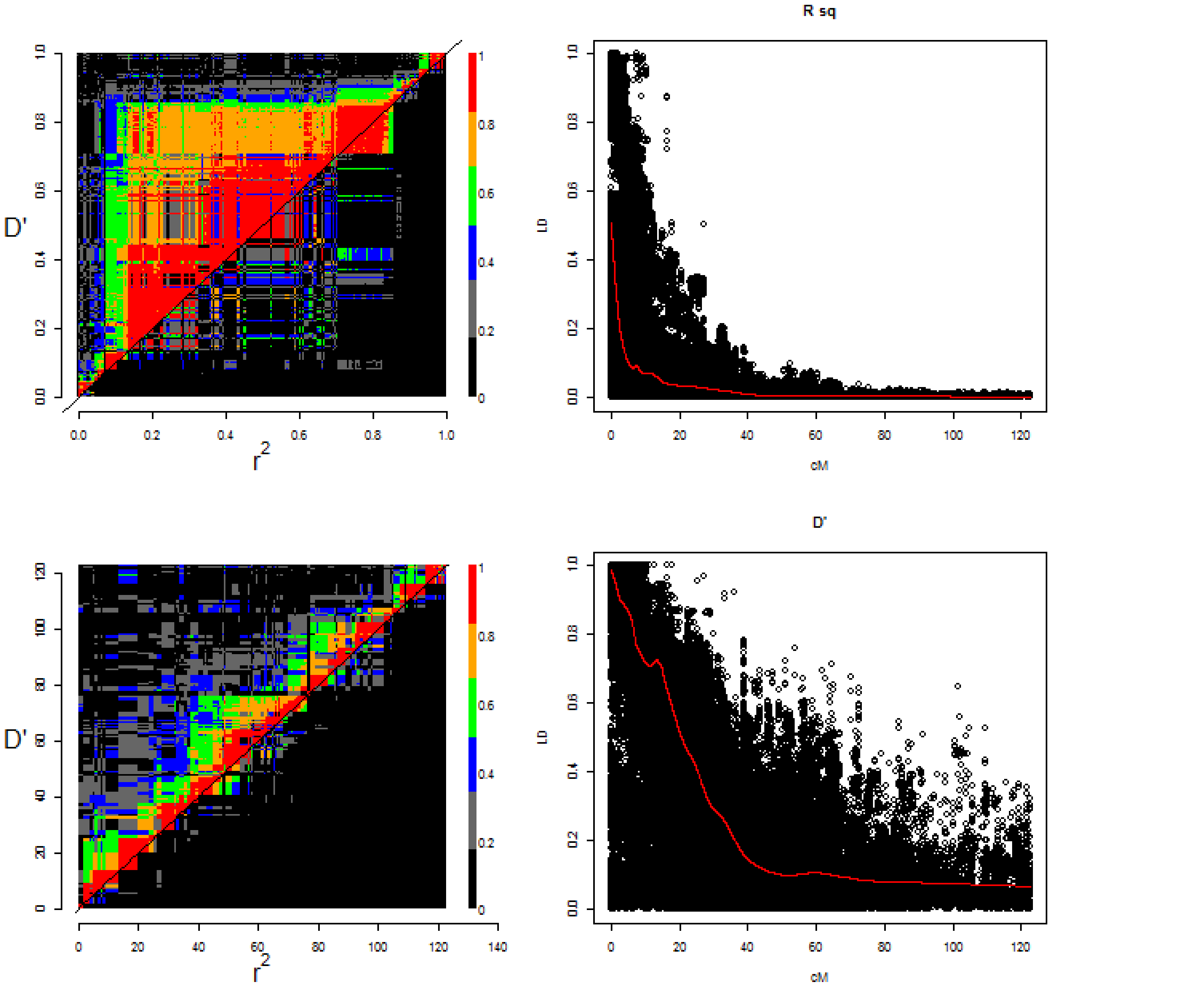

6B

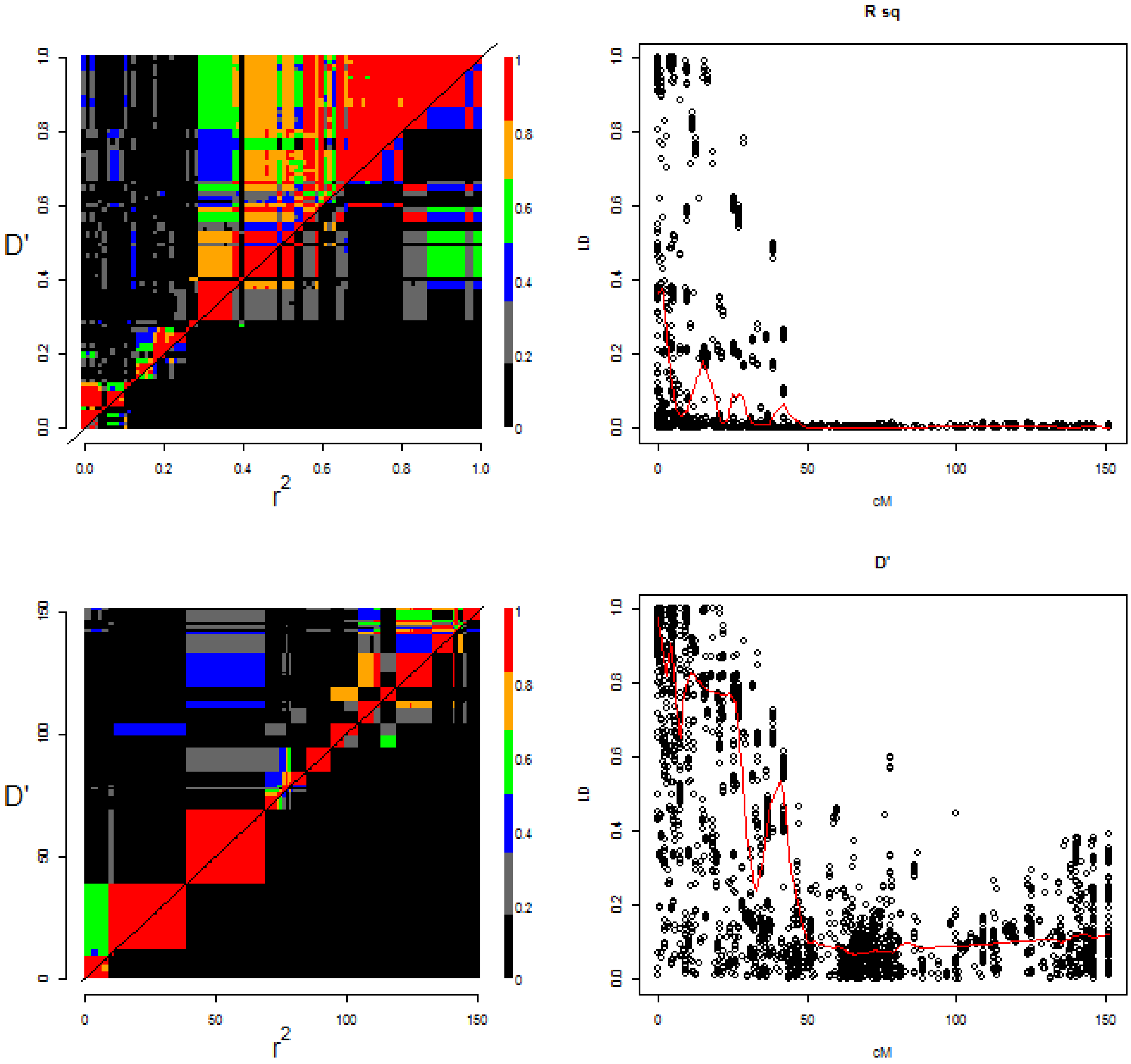

6D

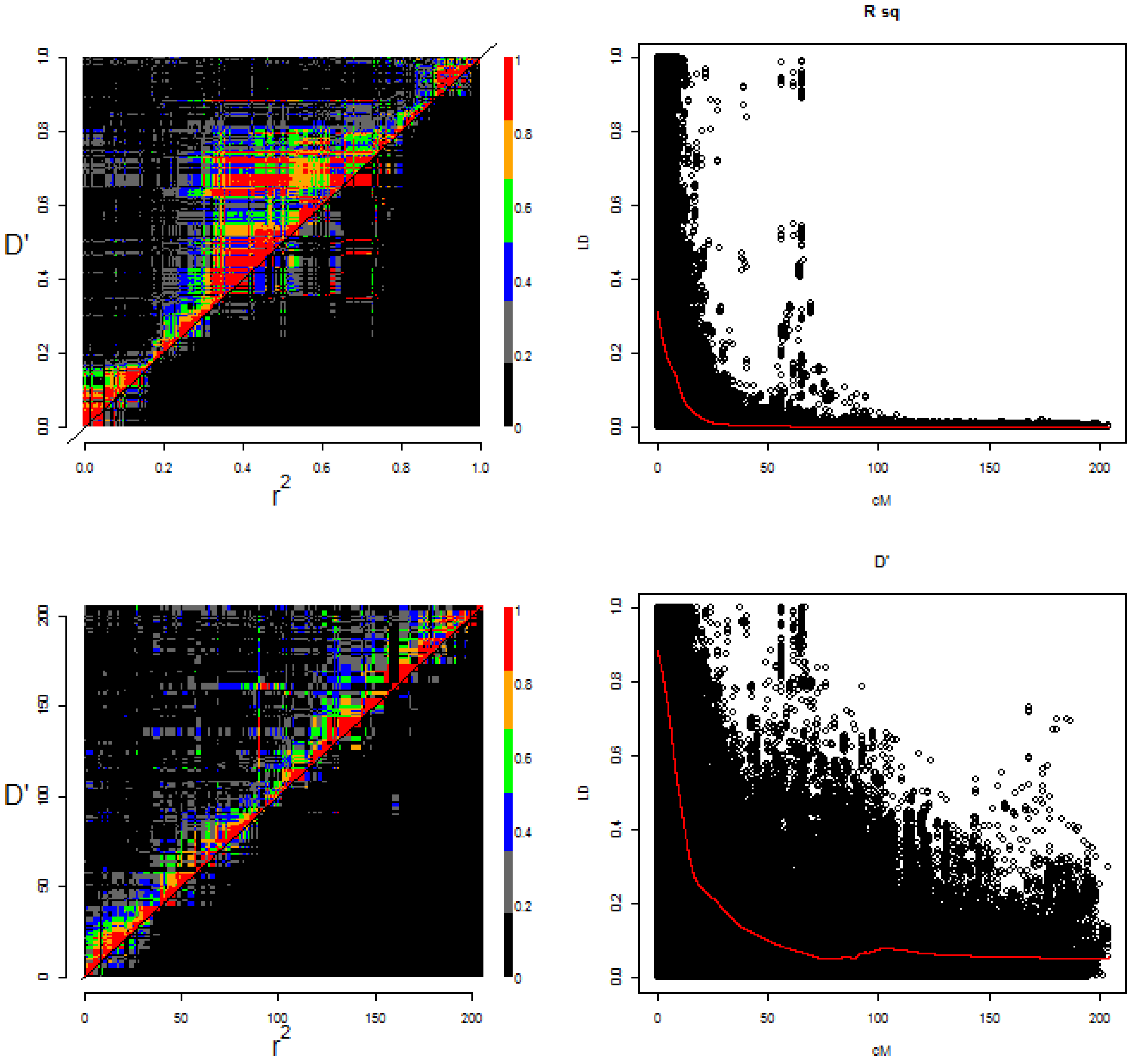

7A

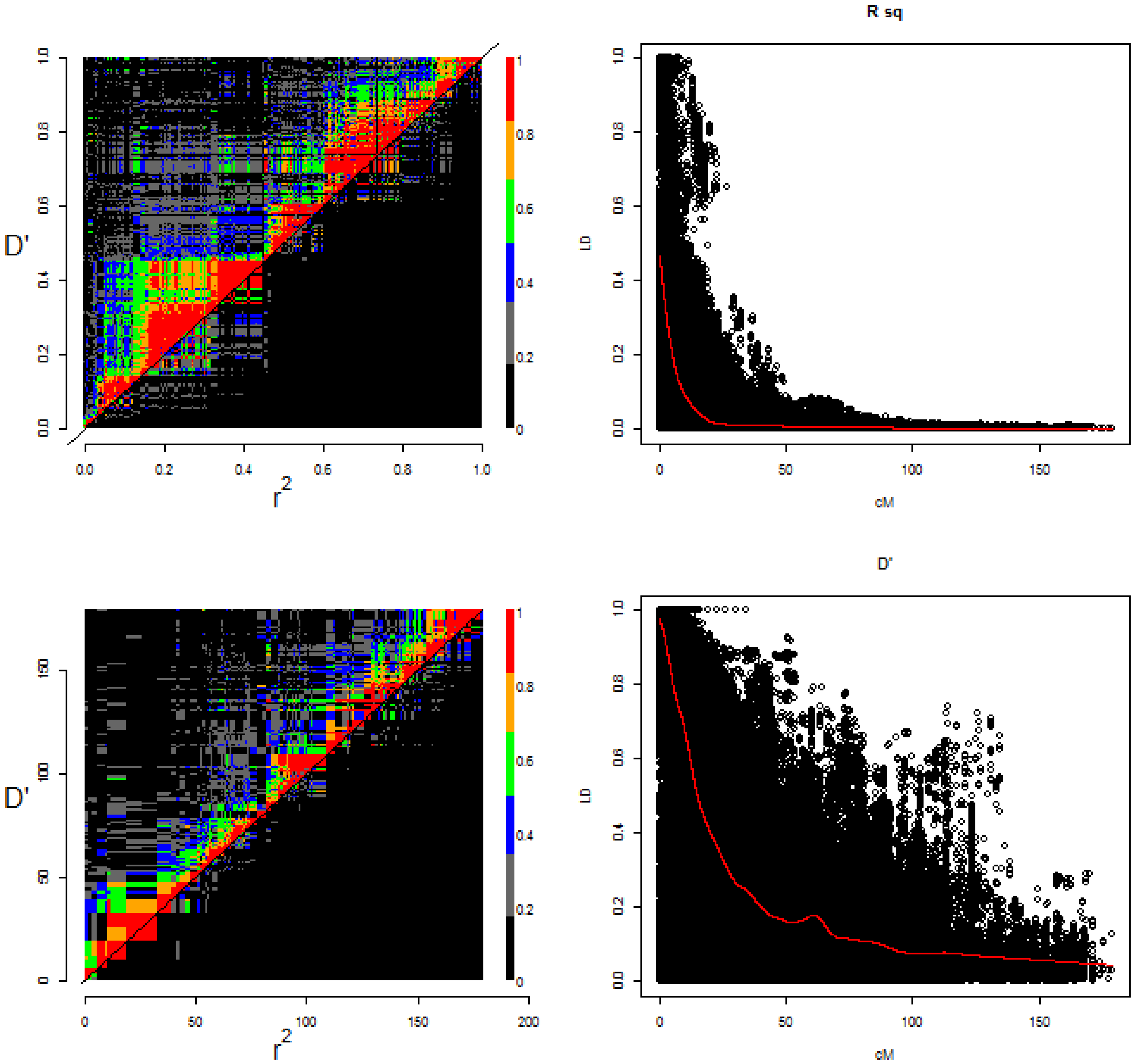

7B

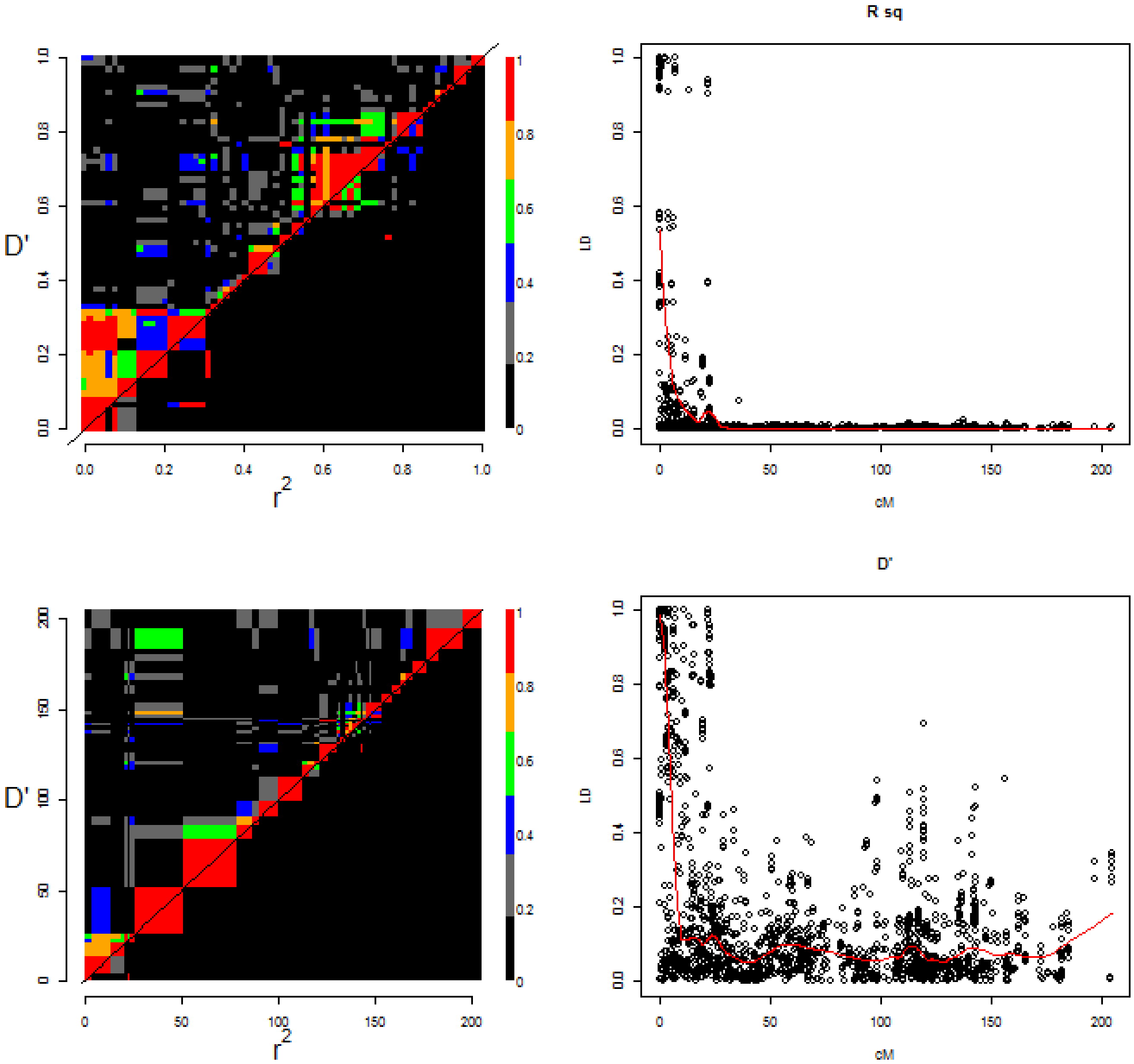

7D
