# Supplementary material for: An Eight-Parent Multiparent Advanced Generation Inter-Cross Population for Winter-Sown Wheat: Creation, Properties, and Validation
Source: G3 (Bethesda). 2014 Sep 1;4(9):1603–10. doi: 10.1534/g3.114.012963 (PMC4169152; doi:10.1534/g3.114.012963)
Supplement: Supporting Information [file supp_4.9.1603_TableS1.pdf]

Table S1 Wheat varieties in the AM panel used to validate the KASP

Variety	BobWhite_C8266_227_TG_5AL	Awn presence/absence
ACCOR	G:G	1
ALTIGO	G:G	1
AMAROK	G:G	1
ARPEGE	G:G	1
BASTIDE	G:G	1
CALISTO	G:G	1
CAPNOR	G:G	1
DI_9714	G:G	1
EUCLIDE	G:G	1
EXOTIC	G:G	1
HURLEY	G:G	1
IENA	G:G	1
INOUI	G:G	1
ISIDOR	G:G	1
NIRVANA	G:G	1
PERNEL	G:G	1
PLETHORE	G:G	1
RODRIGO	G:G	1
RYTMIC	G:G	1
SOISSONS	G:G	1
TILBURI	G:G	1
AARDVARK	T:T	0
ABELE	T:T	0
ABO	T:T	0
ACCESS	T:T	0
ACIENDA	T:T	0
ACIENTO	T:T	0
ADEQUAT	T:T	0
ADMIRAL	T:T	0
AGAMI	T:T	0
AKTEUR	T:T	0
ALBERIC	T:T	0
ALCAZAR	T:T	0
ALCEDO	T:T	0
ALCHEMY	T:T	0
ALDRIC	T:T	0
ALI	T:T	0
ALICANTE	T:T	0
ALIDOS	T:T	0
ALIGRE	T:T	0
ALLANT	T:T	0
ALLEGRO	T:T	0
ALTO	T:T	0
ALTOS	T:T	0
ALTRIA	T:T	0
AMBITION	T:T	0
AMI	T:T	0
ANDAIN	T:T	0
ANDALOU	T:T	0
ANVIL	T:T	0
APACHE	T:T	0
APOLLO	T:T	0
APOSTLE	T:T	0
ARACK	T:T	0
ARBON	T:T	0
ARLEQUIN	T:T	0
ARMENTIERES	T:T	0
ARMINDA	T:T	0
ARON	T:T	0
ASKETIS	T:T	0

ASTRON	T:T	0
ATALANTE	T:T	0
ATLANTIS	T:T	0
ATTLASS	T:T	0
AUBUSSON	T:T	0
AVALON	T:T	0
AVANTAGE	T:T	0
AZIMUT	T:T	0
AZTEC	T:T	0
BALANCE	T:T	0
BALTIMOR	T:T	0
BARON	T:T	0
BAROUDEUR	T:T	0
BATIS	T:T	0
BEAUCHAMP	T:T	0
BEAUFORT	T:T	0
BEAVER	T:T	0
BERMUDE	T:T	0
BISCAY	T:T	0
BOBINO	T:T	0
BOOMER	T:T	0
BOUNTY	T:T	0
BOUQUET	T:T	0
BRANDO	T:T	0
BRENNUS	T:T	0
BRIGADIER	T:T	0
BRIGAND	T:T	0
BRILLIANT	T:T	0
BROCK	T:T	0
BUCHAN	T:T	0
BURMA	T:T	0
BUSTER	T:T	0
BUTEO	T:T	0
CADENZA	T:T	0
CALIF	T:T	0
CAMPARI	T:T	0
CAMPERO	T:T	0
CAPHORN	T:T	0
CAPITOLE	T:T	0
CAPTA	T:T	0
CARDOS	T:T	0
CARENIUS	T:T	0
CARIBO	T:T	0
CENTRUM	T:T	0
CEZANNE	T:T	0
CHAMPION	T:T	0
CHANCELIER	T:T	0
CHARGER	T:T	0
CHATSWORTH	T:T	0
CHIANTI	T:T	0
CLAIRE	T:T	0
CLICK	T:T	0
COLBERT	T:T	0
COMPAL	T:T	0
COMPLIMENT	T:T	0
CONSORT	T:T	0
CONTRA	T:T	0
COPAIN	T:T	0
CORDIALE	T:T	0
CORSAIRE	T:T	0
CORVUS	T:T	0
CRAKLIN	T:T	0
CRISTO	T:T	0

CRITERIUM	T:T	0
CUBUS	T:T	0
DAVIDOC	T:T	0
DEBEN	T:T	0
DEFENDER	T:T	0
DEKAN	T:T	0
DESTINO	T:T	0
DINOSOR	T:T	0
DUCAT	T:T	0
DUKE	T:T	0
EINSTEIN	T:T	0
EKLA	T:T	0
ELEGANT	T:T	0
ELOI	T:T	0
ENORM	T:T	0
EQUINOX	T:T	0
ESTICA	T:T	0
EUREKA	T:T	0
EXALTO	T:T	0
EXCEPT	T:T	0
FESTIN	T:T	0
FEUVERT	T:T	0
FLAIR	T:T	0
FLAME	T:T	0
FLANDERS	T:T	0
FLORENZO	T:T	0
FLORETT	T:T	0
FOLIO	T:T	0
FORBAN	T:T	0
FOURMI	T:T	0
FOURNIL	T:T	0
FRANDOC	T:T	0
FREGATE	T:T	0
FROIDURE	T:T	0
FRUMENT	T:T	0
GALA	T:T	0
GALAHAD	T:T	0
GALAXIE	T:T	0
GAMIN	T:T	0
GARANT	T:T	0
GATSBY	T:T	0
GAUGAIN	T:T	0
GECKO	T:T	0
GENIAL	T:T	0
GLANOR	T:T	0
GLASGOW	T:T	0
GLOCKNER	T:T	0
GOELENT	T:T	0
GRANADA	T:T	0
GRANTA	T:T	0
GREIF	T:T	0
GRISBY	T:T	0
GULLIVER	T:T	0
HAMAC	T:T	0
HATTRICK	T:T	0
HAVEN	T:T	0
HEREWARD	T:T	0
HERMANN	T:T	0
HEROLDO	T:T	0
HOBBIT	T:T	0
HORNET	T:T	0
HUSTLER	T:T	0
HYPERION	T:T	0

IBIS	T:T	0
IGOR	T:T	0
IMPALA	T:T	0
INCISIF	T:T	0
IRIDIUM	T:T	0
ISTABRAQ	T:T	0
JUNIOR	T:T	0
KADOR	T:T	0
KALTOP	T:T	0
LAHERTIS	T:T	0
LANCELOT	T:T	0
LAREDO	T:T	0
LATER	T:T	0
LEIFFER	T:T	0
LIMES	T:T	0
LOUVRE	T:T	0
LUTIN	T:T	0
LYNX	T:T	0
MAGISTER	T:T	0
MAGNUS	T:T	0
MALACCA	T:T	0
MANDUB	T:T	0
MANHATTAN	T:T	0
MAVERICK	T:T	0
MAXYL	T:T	0
MELKIOR	T:T	0
MENDEL	T:T	0
MERCIA	T:T	0
MERKUR	T:T	0
MESSAGER	T:T	0
METEOR	T:T	0
MILVUS	T:T	0
MIRONOVSKAJA	T:T	0
MITCHEL	T:T	0
MOISSON	T:T	0
MURIOT	T:T	0
MYTHOS	T:T	0
NICAM	T:T	0
NOBLET	T:T	0
NORMAN	T:T	0
NOROIT	T:T	0
NUAGE	T:T	0
OAKLEY	T:T	0
OCCITAN	T:T	0
OCTET	T:T	0
OKAPI	T:T	0
OPEN	T:T	0
OPTIDOR	T:T	0
ORATORIO	T:T	0
ORDEAL	T:T	0
OREPI	T:T	0
ORESTIS	T:T	0
ORMIL	T:T	0
ORTOP	T:T	0
ORVANTIS	T:T	0
OURAGAN	T:T	0
PACTOLE	T:T	0
PAINDOR	T:T	0
PAJERO	T:T	0
PARADIS	T:T	0
PAROLI	T:T	0
PASTICHE	T:T	0
PAUILLAC	T:T	0

PEGASOS	T:T	0
PEPITAL	T:T	0
PERICLES	T:T	0
PETRUS	T:T	0
PR-22-R-28	T:T	0
PROTINAL	T:T	0
PULSAR	T:T	0
PYTAGOR	T:T	0
QUATUOR	T:T	0
QUEBON	T:T	0
RAGLAN	T:T	0
RALEIGH	T:T	0
RAPOR	T:T	0
REAPER	T:T	0
RECORD	T:T	0
REMOIS	T:T	0
RESO	T:T	0
RIALTO	T:T	0
RIBAND	T:T	0
RITMO	T:T	0
ROBIGUS	T:T	0
ROMANUS	T:T	0
RUDI	T:T	0
RUMBA	T:T	0
RUSTIC	T:T	0
SAHARA	T:T	0
SAVANNAH	T:T	0
SCHAMANE	T:T	0
SCORE	T:T	0
SENATOR	T:T	0
SEYRAC	T:T	0
SHAMROCK	T:T	0
SHANGO	T:T	0
SKAGEN	T:T	0
SKALMEJE	T:T	0
SOBI	T:T	0
SOCRATES	T:T	0
SOGOOD	T:T	0
SOKRATES	T:T	0
SOLITER	T:T	0
SOLSTICE	T:T	0
SPARK	T:T	0
SPONSOR	T:T	0
STEADFAST	T:T	0
STRIKER	T:T	0
SWINDY	T:T	0
TALENT	T:T	0
TALON	T:T	0
TAMBOR	T:T	0
TANKER	T:T	0
TAPIDOR	T:T	0
TARAS	T:T	0
TARSO	T:T	0
TERRIER	T:T	0
TEXEL	T:T	0
THESEE	T:T	0
TIGER	T:T	0
TIMBER	T:T	0
TOISONDOR	T:T	0
TOP	T:T	0
TORAS	T:T	0
TOREADOR	T:T	0
TORFRIDA	T:T	0

TORONTO	T:T	0
TRANSIT	T:T	0
TREMIE	T:T	0
TRIO	T:T	0
TUAREG	T:T	0
TUKAN	T:T	0
TULSA	T:T	0
TURKIS	T:T	0
URBAN	T:T	0
VALMY	T:T	0
VALORIS	T:T	0
VERCORS	T:T	0
VIRTUE	T:T	0
VIVANT	T:T	0
WASP	T:T	0
WELFORD	T:T	0
WELLINGTON	T:T	0
WESTON	T:T	0
WINNETOU	T:T	0
XI19	T:T	0
ZENTOS	T:T	0
ZOBEL	T:T	0
C-8-14-2	T:T	1
PICADOR	T:T	1
CHATELET	G:G	0
