# Supplementary material for: An Eight-Parent Multiparent Advanced Generation Inter-Cross Population for Winter-Sown Wheat: Creation, Properties, and Validation
Source: G3 (Bethesda). 2014 Sep 1;4(9):1603–10. doi: 10.1534/g3.114.012963 (PMC4169152; doi:10.1534/g3.114.012963)
Supplement: Supporting Information [file supp_4.9.1603_TableS2.pdf]

Table S2 KASP primers for marker BobWhite_c8266_227_TG_5AL. Note, the KASP design assays nucleotides on the opposing strand to the equivalent assay on the Illumina iSelect 90,000 SNP array

KASP Primer	Nucleotide assayed	Predicted phenotype	Primer sequence
Allele X	T	awnless	CGTCCATGGAGTCGTTCCTCAAT
Allele Y	G	awned	GTCCATGGAGTCGTTCCTCAAG
Common			GTGGTACACGTCCGGGAAGAAT
